# Supplementary material for: The Nucleosome Remodeling and Deacetylation Complex Modulates Chromatin Structure at Sites of Active Transcription to Fine-Tune Gene Expression
Source: Mol Cell. 2018 Jul 5;71(1):56–72.e4. doi: 10.1016/j.molcel.2018.06.003 (PMC6039721; doi:10.1016/j.molcel.2018.06.003)
Supplement: Document S1. Figures S1–S6 [file mmc1.pdf]

Molecular Cell, Volume *71*

Supplemental Information

The Nucleosome Remodeling and Deacetylation Complex Modulates Chromatin Structure at Sites of Active Transcription to Fine-Tune Gene Expression

Susanne Bornelöv, Nicola Reynolds, Maria Xenophontos, Sarah Gharbi, Ewan Johnstone, Robin Floyd, Meryem Ralser, Jason Signolet, Remco Loos, Sabine Dietmann, Paul Bertone, and Brian Hendrich

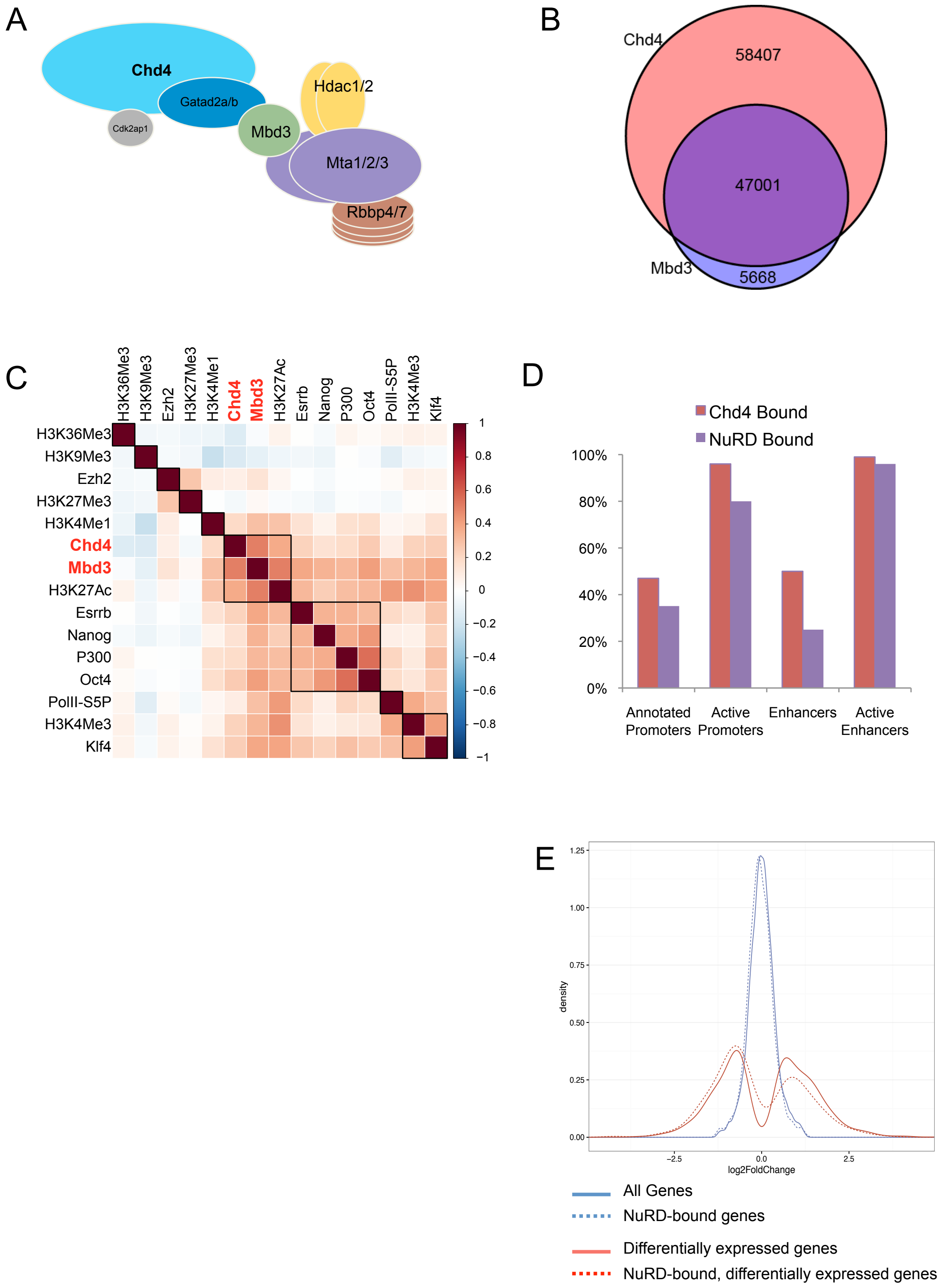

Figure S1. NuRD modulates active transcription. (Related to Figure 1)

A. Schematic of the NuRD complex. Proteins are indicated at approximate stoichiometry. Mbd3 acts to hold together the remodelling subcomplex, containing

Chd4, and the histone deacetylase-containing subcomplex.

B. Overlap of peaks called from Mbd3 or Chd4 ChIP-seq data (blue and pink circles, respectively) in mouse ES cells. Numbers of peaks corresponding to Chd4-only, Mbd3 and Chd4, or Mbd3-only are indicated.

C. Correlation between ChIP peaks for the indicated histone modifications and transcription factors in 2i/LIF. Datasets are ordered by unsupervised clustering. Boxes indicate the highest correlations. Datasets used are listed in the STAR Methods.

D. The proportion of promoters and enhancers containing a Chd4- or NuRD-bound (defined as Mbd3- and Chd4-bound) site are plotted.

E. Changes in gene expression in *Mbd3*-null vs wild-type ES cells. Red lines indicate genes that exhibit a significant change in mutant cells, blue lines indicate no significant change. Genes showing a ChIP-seq peak for Mbd3 and Chd4 between -2 Kb and + 0.5 Kb of the annotated TSS are indicated as dotted lines. y-axis: kernel density estimation; x-axis: $\log_2$ fold change (KO/WT).

Bornelöv et al. Figure S2

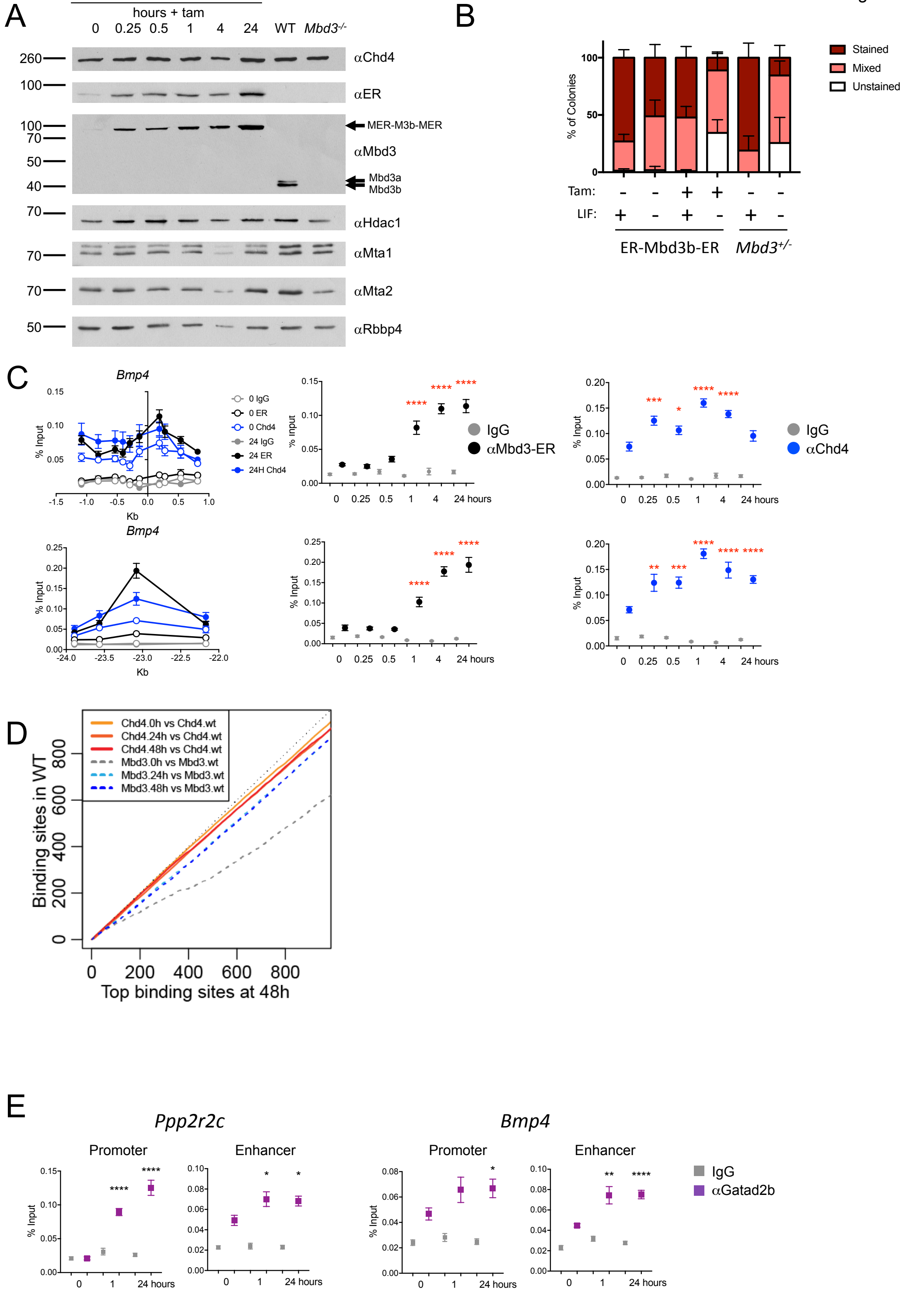

Figure S2. Further characterisation of the Mbd3 inducible system (Related to Figure 1).

A. Nuclear extracts obtained from Mbd3 inducible ES cells at different times after tamoxifen addition, from wild type ES cells (WT), or from *Mbd3*-null ES cells (*Mbd3*$^{-/-}$)

were probed with antibodies indicated at right. In the α-Mbd3 panel the location of the Mer-Mbd3b-Mer transgene is indicated with an arrow, as are the locations of endogenous Mbd3 isoforms in wild type cells. Protein sizes are shown at left in kilodaltons.

B. Mbd3b inducible cells with or without tamoxifen (+ or - TAM) and Mbd3(+/-) ES cells were plated in self-renewing (Serum+LIF) or differentiation conditions (Serum without LIF) at clonal density and allowed to grow for five days prior to staining for alkaline phosphatase activity and scored blind. The percentage of unstained, mixed, or fully stained colonies is shown. Mean ± SD is plotted where N = 3-12 per condition.

C. Left: ChIP-qPCR for Mbd3 (black lines), Chd4 (blue lines) and IgG control (grey lines) across the promoter (top) and an enhancer (bottom) for *Bmp4*. N ≥ 3 biological replicates. X-axes show locations relative to the annotated transcription start site of *Bmp4*. ChIP-qPCR at the peak of the Mbd3 ChIP signal was plotted across a time course of tamoxifen addition (middle and right panels). **** P<0.0001, *** P<0.001, ** P<0.01, * P<0.05) using a two-tailed t-test. N ≥ 3 biological replicates.

D. Validation of Mbd3 and Chd4 binding site recovery after Mbd3 induction in the inducible cell line. The cumulative overlap between binding sites 0h, 24h, or 48h after Mbd3 induction and in WT is shown. All binding sites were ranked by their p-value and the 20,000 most significant sites in WT were considered for the overlap calculations.

E. ChIP-qPCR for Gatad2b across the tamoxifen induction time course at single points at the indicated features. **** P<0.0001, *** P<0.001, ** P<0.01, * P<0.05) using a two-tailed t-test. N ≥ 3 biological replicates.

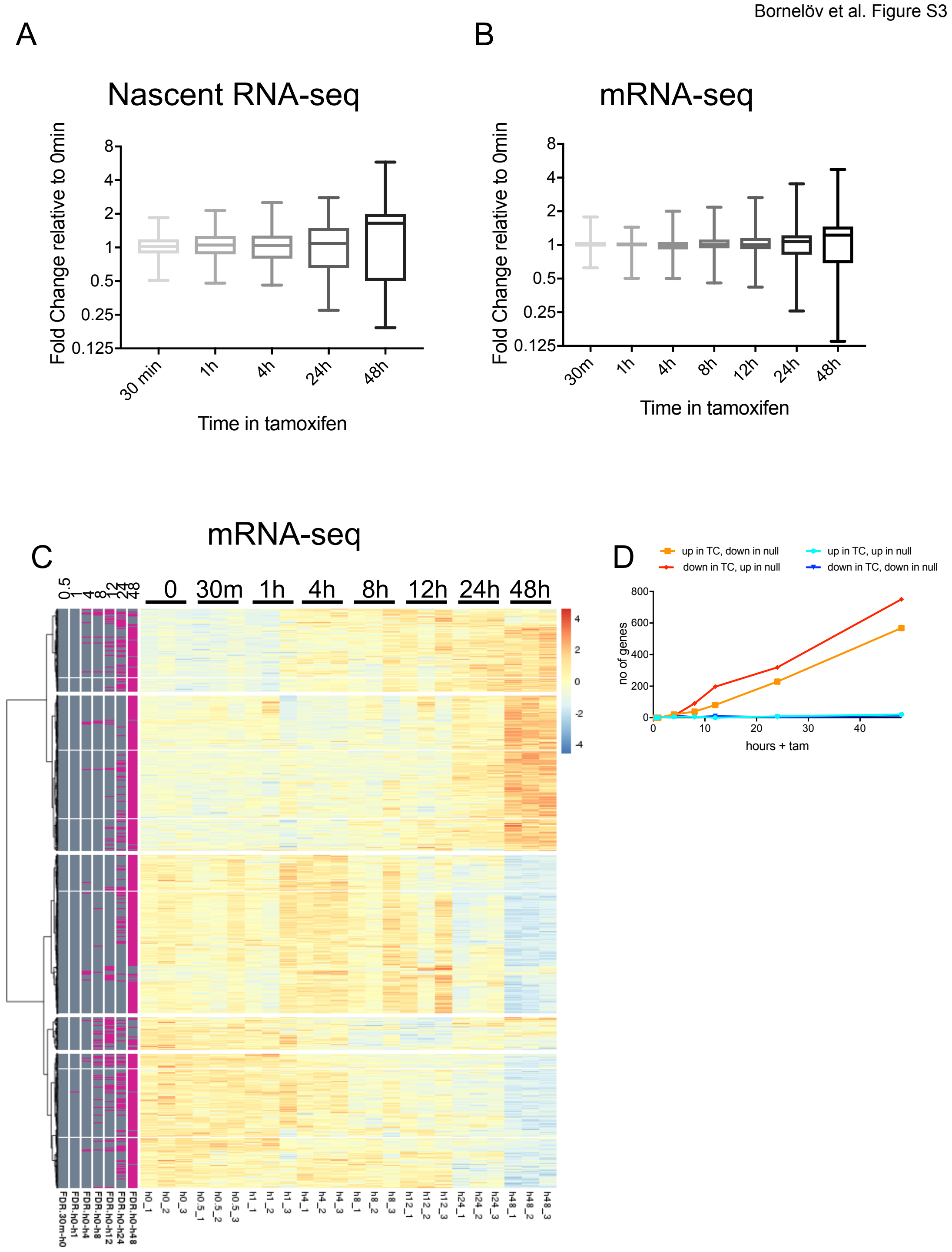

Figure S3. Gene expression changes in the Mbd3 inducible system (Related to Figure 1).

A and B: Fold change in gene expression plotted for all genes showing significant change (P<0.01, |FC|>2) at 48 hours of tamoxifen treatment relative to untreated cells for nascent RNA-seq (A; N = 816) and for mRNA-seq (B; N = 2210).

C. Unsupervised clustering of gene expression changes over indicated times of the time course of tamoxifen exposure as measured by mRNA-seq. Data for each of three biological replicates are shown. Genes exhibiting a significant change relative to time 0 are shaded pink in the columns at left.

D Comparison of changes in gene expression in the Mbd3 induction time course with changes seen in steady-state Mbd3-null ES cells.

Bornelöv et al. Figure S4

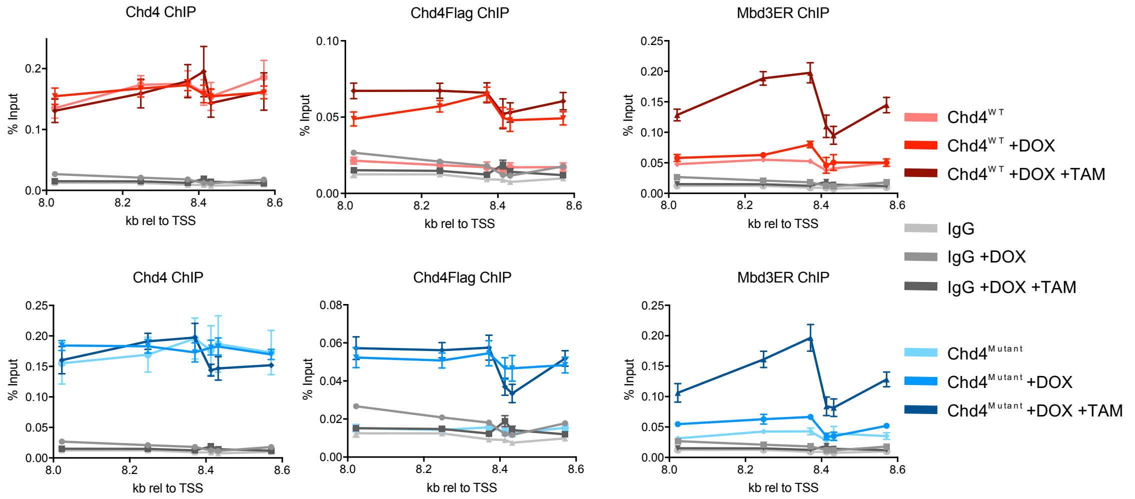

Figure S4. Induced Chd4 protein associates with chromatin (Related to Figure 4).

ChIP-qPCR across the *Ppp2r2c* enhancer for Chd4 (left), anti-FLAG, which recognises the Chd4 transgenic protein (middle) and for the induced Mbd3 protein (right) in ES cells harbouring the wild type Chd4 transgene (Chd4^{WT}) or the ATPase mutant Chd4 transgene (Chd4^{Mutant}). ChIP was performed in uninduced cells and in cells induced with DOX only or DOX and TAM. N = 3-6 biological replicates.

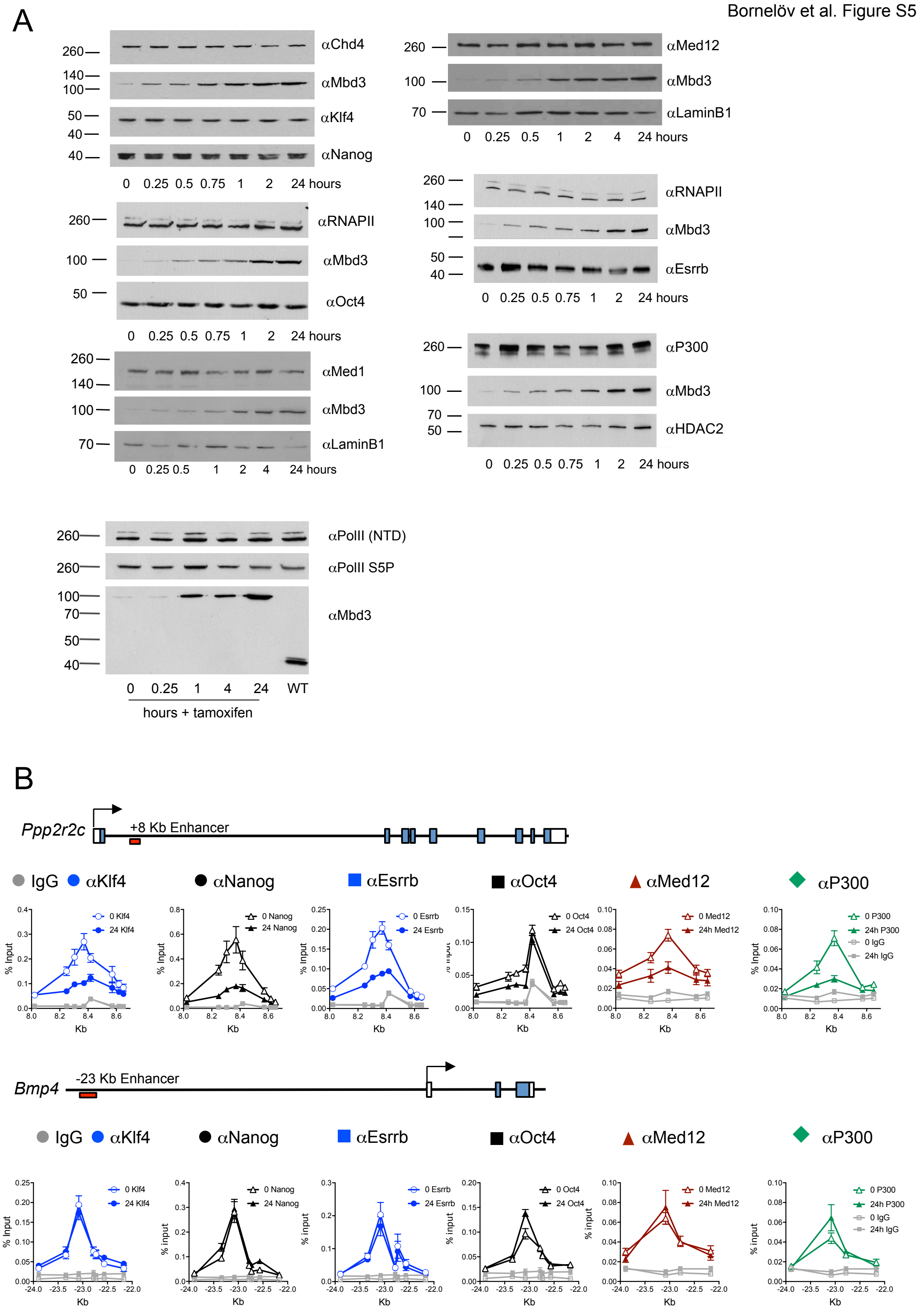

Figure S5. Transcription factors behaviour across the Mbd3 induction time course (Related to Figure 5).

A. Nuclear extracts obtained from Mbd3 inducible ES cells at different times across a tamoxifen addition time course were probed with indicated antibodies. Each set of panels was taken from the same gel. Protein sizes are shown at left in kilodaltons. Full western blot images are available in Supplemental Data.

B. ChIP-qPCR for indicated proteins across the *Ppp2r2c* enhancer or the *Bmp4* enhancer at 0 or 24 hours of tamoxifen exposure. A schematic of each gene is shown above the ChIP-qPCR panels. Mean ± SEM is plotted for all points. N ≥ 3 biological replicates.

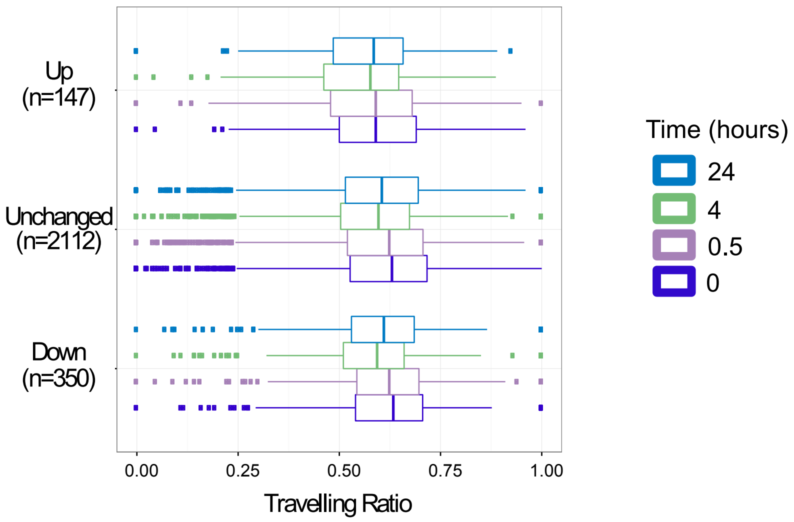

Figure S6. Travelling ratios as a function of changes in gene expression (Related to Figure 6).

Travelling ratios for genes showing increased (Up), decreased (Down) or no change (Unchanged) in the Mbd3 induction time course are plotted at indicated times after tamoxifen addition.
