# Supplementary material for: The Nucleosome Remodeling and Deacetylation Complex Modulates Chromatin Structure at Sites of Active Transcription to Fine-Tune Gene Expression
Source: Mol Cell. 2018 Jul 5;71(1):56–72.e4. doi: 10.1016/j.molcel.2018.06.003 (PMC6039721; doi:10.1016/j.molcel.2018.06.003)
Supplement: Document S2. Article plus Supplemental Information [file mmc6.pdf]

Article

Molecular Cell

The Nucleosome Remodeling and Deacetylation Complex Modulates Chromatin Structure at Sites of Active Transcription to Fine-Tune Gene Expression

Graphical Abstract

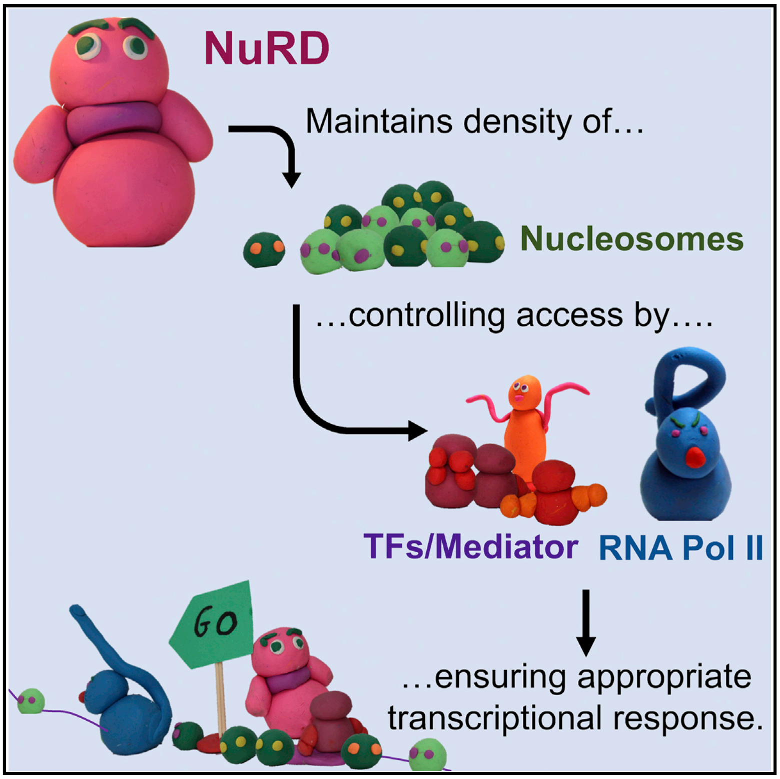

Authors

Susanne Bornelöv, Nicola Reynolds, Maria Xenophontos, ..., Sabine Dietmann, Paul Bertone, Brian Hendrich

Correspondence

bertone@stemcells.cam.ac.uk (P.B.), bdh24@cam.ac.uk (B.H.)

In Brief

Bornelöv et al. define how NuRD, an abundant chromatin remodeling complex, fine-tunes gene expression. NuRD controls nucleosome positioning across regulatory elements genome-wide, controlling access of DNA-binding proteins to enhancers and promoters. This resetting of the transcription factor repertoire at regulatory elements restricts expression from some loci and induces transcription at others.

Highlights

- NuRD increases nucleosome density, expelling TFs or inhibiting recruitment
- NuRD displaces RNA Pol II from TSSs, reducing nascent transcription
- Local gains in TF and Mediator occupancy can be indirect effects of NuRD activity
- Resetting protein binding at regulatory elements can promote or suppress transcription

Bornelöv et al., 2018, Molecular Cell *71*, 56–72
July 5, 2018 © 2018 The Author(s). Published by Elsevier Inc.
https://doi.org/10.1016/j.molcel.2018.06.003

CellPress

Molecular Cell
Article

The Nucleosome Remodeling and Deacetylation Complex Modulates Chromatin Structure at Sites of Active Transcription to Fine-Tune Gene Expression

Susanne Bornelöv,[1,4] Nicola Reynolds,[1,4] Maria Xenophontos,[1,2] Sarah Gharbi,[1] Ewan Johnstone,[1,2] Robin Floyd,[1] Meryem Ralser,[1] Jason Signolet,[1,3] Remco Loos,[1,2] Sabine Dietmann,[1] Paul Bertone,[1,2,*] and Brian Hendrich[1,3,5,*]
[1]Wellcome-MRC Stem Cell Institute, University of Cambridge, Gleeson Building, Tennis Court Road, Cambridge CB2 1QR, UK
[2]European Bioinformatics Institute, European Molecular Biology Laboratory (EMBL), Wellcome Trust Genome Campus, Cambridge CB10 1SD, UK
[3]Department of Biochemistry, University of Cambridge, Tennis Court Road, Cambridge CB2 1QR, UK
[4]These authors contributed equally
[5]Lead Contact
*Correspondence: bertone@stemcells.cam.ac.uk (P.B.), bdh24@cam.ac.uk (B.H.)
https://doi.org/10.1016/j.molcel.2018.06.003

SUMMARY

Chromatin remodeling complexes play essential roles in metazoan development through widespread control of gene expression, but the precise molecular mechanisms by which they do this *in vivo* remain ill defined. Using an inducible system with fine temporal resolution, we show that the nucleosome remodeling and deacetylation (NuRD) complex controls chromatin architecture and the protein binding repertoire at regulatory regions during cell state transitions. This is primarily exerted through its nucleosome remodeling activity while deacetylation at H3K27 follows changes in gene expression. Additionally, NuRD activity influences association of RNA polymerase II at transcription start sites and subsequent nascent transcript production, thereby guiding the establishment of lineage-appropriate transcriptional programs. These findings provide a detailed molecular picture of genome-wide modulation of lineage-specific transcription by an essential chromatin remodeling complex as well as insight into the orchestration of molecular events involved in transcriptional transitions *in vivo*.

INTRODUCTION

The nucleosome remodeling and deacetylation (NuRD) complex is an abundant, highly conserved multiprotein chromatin remodeler initially defined as a transcriptional repressor (Tong et al., 1998; Wade et al., 1998; Xue et al., 1998; Zhang et al., 1998). NuRD activity facilitates cell fate transitions in a range of different organisms and developmental contexts (Signolet and Hendrich, 2015). The complex combines two enzymatic activities in the form of class I lysine deacetylation, encoded by the Hdac1 and 2 proteins, with the Swi/Snf-type ATPase and nucleosome remodeling of Chd4. Additionally, the complex contains histone chaperone proteins Rbbp4 and Rbbp7, one of the zinc-finger proteins Gatad2a or Gatad2b, two MTA proteins (Mta1, Mta2, and/or Mta3), Cdk2ap1, and Mbd2 or Mbd3 (Allen et al., 2013; Kloet et al., 2015; Mohd-Sarip et al., 2017). With the exceptions of the histone deacetylase proteins and Rbbp4 and 7, these proteins have been found only within the NuRD complex.

NuRD is an abundant chromatin-associated protein complex, and its components have been reported to physically interact with a wide repertoire of transcription factors. Indeed, a few of these have been shown to recruit NuRD to specific DNA sequences to influence transcription of individual target genes (e.g., Aguilera et al., 2011; Hong et al., 2005; Liang et al., 2017). Genome-wide mapping of chromatin binding patterns of NuRD components has shown them to occupy virtually all active enhancers and promoters in a variety of cell types (Figure S1; de Dieuleveult et al., 2016; Günther et al., 2013; Miller et al., 2016; Shimbo et al., 2013; Stevens et al., 2017). Rather than being recruited to every active enhancer and promoter by individual sequence-specific transcription factors, these global localization data are more consistent with a model in which NuRD has a general affinity for open chromatin or possibly for the transcription machinery. Sequence-specific transcription factors might then act to locally increase NuRD concentrations at individual target loci.

The Mbd3 protein was originally identified by sequence similarity to the methyl-CpG binding protein Mecp2, but the putative methyl-CpG binding domain of Mbd3 contains mutations that prevent binding to methylated DNA (Hendrich and Bird, 1998; Saito and Ishikawa, 2002). Mbd3 is required for lineage commitment of pluripotent cells and is essential for early mammalian development (Signolet and Hendrich, 2015). Notably, the methyl-CpG binding domain-like region of Mbd3 is dispensable in normal differentiation and development. Recent structural work has found that Mbd3 physically links two biochemical and functional NuRD subcomplexes: a remodeling subcomplex containing Chd4, Gatad2a/b, and Cdk2ap1 and a histone deacetylase subcomplex containing the HDAC, MTA, and RBBP proteins (Figure S1A; Low et al., 2016; Zhang et al., 2016). Mbd3

 © 2018 The Author(s). Published by Elsevier Inc.
This is an open access article under the CC BY license (http://creativecommons.org/licenses/by/4.0/).

acts as a molecular bridge between these subcomplexes and thus maintains the structural integrity of NuRD.

Mbd2 and Mbd3 are mutually exclusive components of NuRD, and Mbd2/NuRD and Mbd3/NuRD have been found to exhibit distinct but overlapping functions (Günther et al., 2013; Hendrich et al., 2001; Le Guezennec et al., 2006). In mouse embryonic stem cells (ESCs), Mbd3/NuRD activity modulates the transcription of pluripotency-associated genes, maintaining expression within a range that allows cells to effectively respond to differentiation signals (Reynolds et al., 2012a). Despite profound developmental defects, Mbd3 deficiency in ESCs results in only moderate gene expression changes, with the majority of genes changing by less than two-fold (Miller et al., 2016; Reynolds et al., 2012b). Rather than turning genes on or off, Mbd3/NuRD activity serves to fine-tune gene expression in ESCs. Although this amounts to many small transcriptional changes, the cumulative effect of this is nevertheless an acute phenotype: the inability of pluripotent cells to undergo lineage commitment.

How the two distinct enzymatic activities of NuRD individually influence the transcriptional machinery is not clear. NuRD activity results in loss of H3K27 acetylation, providing a substrate for PRC2-mediated trimethylation at some sites (Reynolds et al., 2012b). The nucleosome remodeling activity of Chd4 generally increases nucleosome density at target sites and facilitates lineage commitment through control of gene expression probability (de Dieuleveult et al., 2016; Liang et al., 2017; Morris et al., 2014; Moshkin et al., 2012; O'Shaughnessy-Kirwan et al., 2015), but exactly how Chd4-depdendent nucleosome remodeling mechanistically impacts transcription is unknown. A much more detailed understanding of the relative functions of these two activities is required to understand how transcription is so precisely controlled in developmental contexts.

In this study, we set out to determine how NuRD fine-tunes gene expression. Using an inducible ESC system with fine temporal resolution, we show that transcriptional control by the NuRD complex is primarily exerted through its chromatin remodeling activity. NuRD acts at regulatory sequences to increase nucleosome density, which in turn influences the ability of transcription factors, coactivators, and RNA polymerase II to associate with those sequences. We further demonstrate that NuRD-dependent nucleosome rearrangements are used by cells to control protein access to enhancers during lineage commitment. This shows how the dual enzymatic activities of one chromatin-modifying complex impact the transcription machinery to precisely control transcription levels during cell state transitions.

RESULTS

NuRD Fine-Tunes Active Transcription in Mouse ESCs

Two NuRD component proteins, the ATP-dependent helicase Chd4 and the scaffold protein Mbd3, associate with chromatin extensively in ESCs (Figures S1A and S1B). Mbd3 occupancy is almost completely coincident with Chd4, consistent with the presence of Mbd3 exclusively within the NuRD complex (Figure S1B). In contrast, less than half of all Chd4 sites are also bound by Mbd3, in line with data showing that Chd4 also functions independently of NuRD (O'Shaughnessy and Hendrich, 2013; O'Shaughnessy-Kirwan et al. 2015). Binding of both Chd4 and Mbd3 correlates strongly with indicators of active promoters and enhancers, such as H3K27Ac, H3K4Me1, H3K4Me3, P300, and the initiating form of RNA polymerase II (PolII-S5P; Figure S1C), as well as with pluripotency-associated transcription factors Oct4, Nanog, Esrrb, and Klf4. In contrast, NuRD component binding is anti-correlated with a mark of silent chromatin (H3K9Me3) and with another histone modification deposited across transcribed gene bodies (H3K36Me3). Weak correlation is seen with Ezh2 and trimethylated H3K27, consistent with NuRD cooperating with PRC2 at a subset of loci in ESCs (Reynolds et al., 2012b). NuRD and Chd4 co-occupy nearly all active enhancers and active promoters in ESCs (Figure S1D), indicating that NuRD is tightly associated with the active transcriptional machinery. Despite the widespread presence of NuRD on sites of active transcription, loss of the Mbd3 component (and dissolution of Mbd3/NuRD) results in only moderate changes in gene expression (Figure S1E). Taken together, these data demonstrate that Mbd3/NuRD associates with sites of active transcription and functions not to turn genes on or off but rather to fine-tune gene expression in ESCs.

An Inducible Mbd3 System Allows Induction of NuRD Activity at High Temporal Resolution

We wished to understand how the enzymatic activities of the NuRD complex regulate gene expression. We therefore sought to measure the consequences of acute NuRD recruitment on chromatin structure and transcription of NuRD-responsive genes. We employed a system that allowed us to restore NuRD activity to a cell in which the functional complex is lacking and subsequently monitored the impact on chromatin and transcription over time. The "b" isoform of the Mbd3 protein (Hendrich and Bird, 1998) fused to mouse estrogen receptor (ER) domains at both the N and C termini was expressed in *Mbd3*-null ESCs (Figure 1A). The "b" isoform of Mbd3 lacks the N-terminal half of the methyl-CpG binding domain-like region and is the predominant isoform in mouse ESCs (Figures 1B and S2A). In the absence of tamoxifen, the ER-Mbd3b-ER protein is confined to the cytoplasm, and cells adopt an *Mbd3*-null phenotype, in that they are resistant to differentiation (Figures 1A and S2B; Reynolds et al., 2012b). In the absence of nuclear Mbd3, the NuRD component proteins Mta3 and Gatad2b show reduced protein stability (Figure 1B). Although Chd4 remains associated with existing Gatad2b protein in the nucleus, there is little detectable interaction between Chd4 and components of the NuRD histone deacetylase subcomplex, such as Mta1 or Hdac1 (Figure 1C). Upon introduction of tamoxifen to the culture media, ER-Mbd3b-ER rapidly translocates into the nucleus, restoring the stability of NuRD complex components (Figures 1B and S2C) and the biochemical interactions between Chd4 and components of the deacetylase subcomplex (Figure 1C). Differentiation competence is also re-established (Figure S2B), indicating the restoration of NuRD function. Thus, our system allows the selective induction of NuRD formation and function in a highly controllable manner.

The induced Mbd3 protein was detectable by chromatin immunoprecipitation (ChIP) on known NuRD target sequences

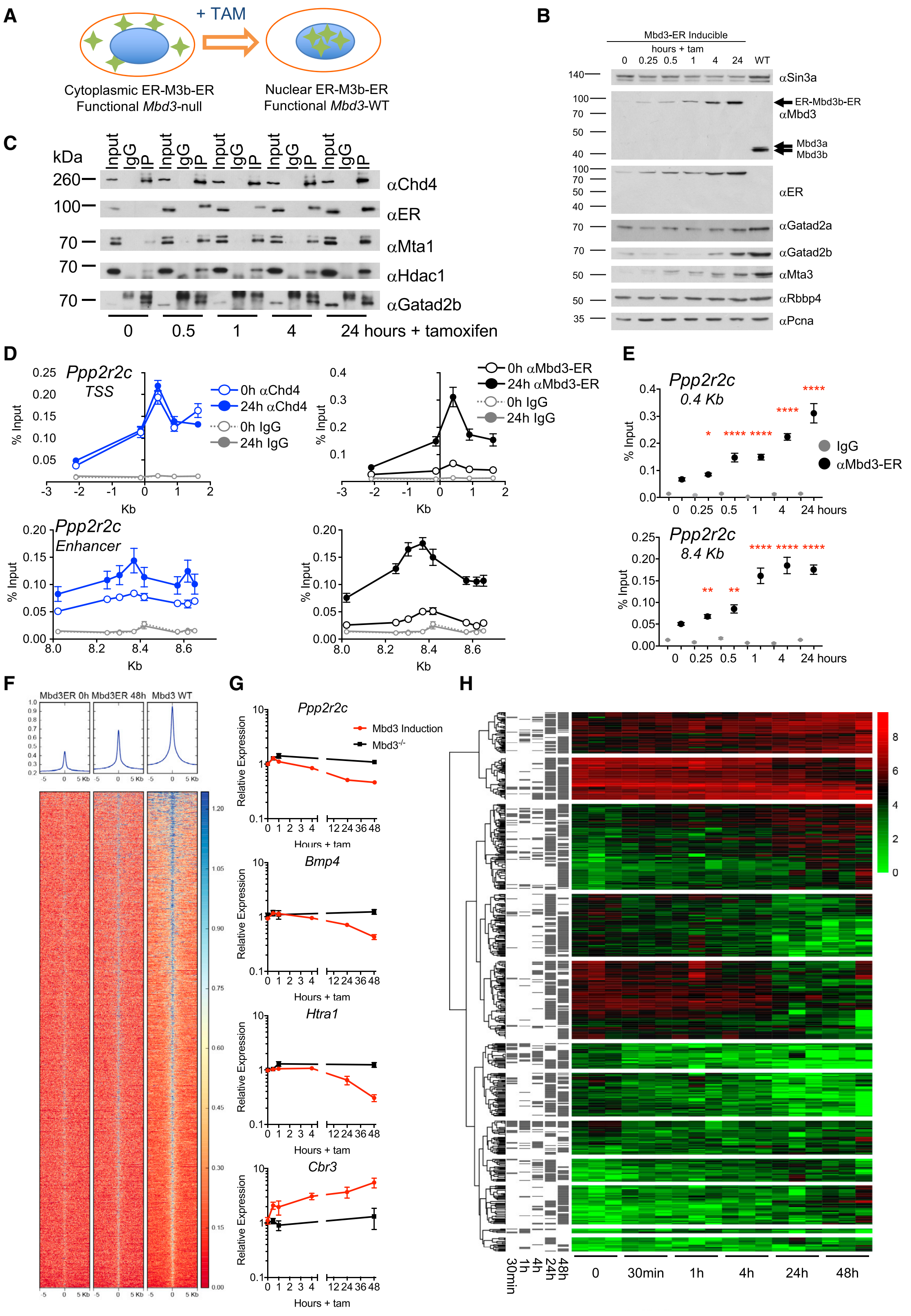

(legend on next page)

between 15 and 30 min after tamoxifen addition, where enrichment continued to increase over 48 hr (Figures 1D, 1E, and S2C). Binding at both 24 hr and 48 hr is highly correlated with Mbd3 ChIP sequencing (ChIP-seq) signal measured in wild-type cells, demonstrating that ER-Mbd3b-ER is properly targeted to chromatin in our induction system (Figures 1F and S2D). NuRD component protein Gatad2b showed increased enrichment at NuRD target sites within 1 hr of tamoxifen addition, consistent with the assembly and recruitment of the NuRD complex to chromatin as a consequence of Mbd3 induction (Figure S2E). Levels of Chd4 enrichment at some, but not all, Mbd3 target loci increased over this time course (Figures 1D and S2C). However, the presence of Mbd3 had no detectable impact on the distribution of Chd4-bound sites (Figure S2D), indicating that Chd4 is able to bind chromatin independently of Mbd3 and the rest of the NuRD complex, though Mbd3 may serve to stabilize Chd4-chromatin interaction.

Restoration of NuRD activity had a rapid impact on gene expression. Coincident with NuRD complex formation and association to chromatin (Figures 1C and 1E), changes in the levels of nascent transcripts were detectable within 30 min of Mbd3 induction and increased steadily through 48 hr (Figures 1G and 1H). Distinct classes of transcriptional changes were evident: both increases and decreases in expression ≤ 4 hr after Mbd3 induction or after a lag of 1 or 2 days. As was seen in steady-state conditions, the impact of NuRD induction on transcriptional output was modest, with the majority of genes changing by less than 2-fold even by 48 hr (Figures 1G, 1H, and S3A–S3C). This observation is consistent with a transcriptional modulatory function for the complex, rather than with it simply turning genes on or off. Persistent and significant changes in steady-state mRNA levels were first detectable by total RNA-seq 4–8 hr post-tamoxifen addition and also increased steadily through 48 hr (Figures S3B and S3C). Gene expression changes seen upon Mbd3 induction were consistent with those observed in *Mbd3*$^{-/-}$ ESCs (Figure S3D). The timescale for induction of NuRD complex formation, recruitment to chromatin, and subsequent changes in transcriptional activity thus provides a means to probe the molecular changes that underlie NuRD-dependent gene regulation.

Histone H3 Lysine 27 Acetylation Changes Follow but Do Not Precede NuRD-Dependent Transcriptional Changes

The NuRD complex comprises two enzymatic activities: class I protein deacetylation in the Hdac1 and 2 proteins and nucleosome remodeling through Chd4. We reasoned that NuRD would affect its transcriptional modulation through one or both of these two activities. Considering the former, we previously showed that acetylation of histone H3 lysine 27 (H3K27Ac), a mark generally associated with active transcription, is anticorrelated with NuRD activity (Reynolds et al., 2012b). We therefore investigated whether induction of NuRD would have an immediate impact on levels of H3K27 acetylation at genes showing a transcriptional response to NuRD induction.

Genes for which a change in mRNA levels was detectable within 8 hr of tamoxifen addition showed no significant differences in H3K27Ac within this time frame (repressed or activated early; Figures 2A and 2B, left panels). A change in H3K27Ac levels at these genes was detectable 48 hr after tamoxifen addition, commensurate with the direction of transcriptional change (Figures 2A and 2B, middle panels). Genes at which a change in mRNA levels was detectable only 24–48 hr after tamoxifen addition showed no significant change in H3K27Ac levels even after 48 hr (repressed or activated late; Figures 2A and 2B, right panels). We followed alterations in H3K27 acetylation at finer temporal resolution by ChIP-qPCR at two NuRD-repressed genes (*Ppp2r2c* and *Htra1*; Figure 1G). H3K27Ac levels at both promoters decreased only after transcription had already been affected, showing slight initial increases within four hours post-tamoxifen exposure followed by a gradual decrease through 48 hr (Figure 2C). Genome-wide, levels of H3K27Ac showed a slight decrease at NuRD-bound loci after four hours, but initial levels were restored by 24 hr (Figure 2D). Changes in H3K27 acetylation are therefore unlikely to drive the acute transcriptional changes observed after NuRD induction but rather may act to reinforce them.

NuRD Induction Rapidly Induces Changes in Chromatin Structure

The other enzymatic activity contained within NuRD is the ATPase-dependent nucleosome remodeling capacity of Chd4.

Figure 1. Mbd3 Induction Restores NuRD Activity to *Mbd3*-Null ESCs

(A) Model of the induction system: *Mbd3*-null ESCs (left) contain ER-Mbd3b-ER (green diamonds, ER-M3b-ER) in the cytoplasm. Upon addition of tamoxifen, ER-M3b-ER enters the nucleus.

(B) Nuclear extracts from Mbd3-inducible ESCs at different times after tamoxifen addition or from wild-type ESCs (WT) were probed with antibodies indicated at right. In the α-Mbd3 panel, the location of the ER-Mbd3b-ER transgene is indicated with an arrow, as are the locations of endogenous Mbd3 isoforms in WT cells. Sin3A and Pcna act as loading controls. Protein sizes are shown at left in kilodaltons.

(C) Chd4 was immunoprecipitated from nuclear protein extracts across tamoxifen induction time course and probed with antibodies indicated at right. IgG, immunoglobulin G (IgG) control; Input, 10% input; IP, Chd4 immunoprecipitation. Protein sizes are shown at left in kilodaltons.

(D) ChIP-qPCR for Chd4 (blue lines), Mbd3 (black lines), and IgG (gray lines) across the promoter and an enhancer of NuRD target gene *Ppp2r2c* at 0 and 24 hr of tamoxifen treatment. x axes show locations relative to the annotated transcription start site of *Ppp2r2c*. N $\geq$ 3 biological replicates. See also Figure S2C.

(E) ChIP-qPCR at the peak of Mbd3 ChIP signal in (D) plotted across a time course of tamoxifen addition. Significant enrichment of Mbd3 relative to no tamoxifen occurs from 15 min onward (****$p < 0.0001$, **$p < 0.01$, and *$p < 0.05$ using a two-tailed t test). N $\geq$ 3 biological replicates.

(F) Input-normalized ChIP signal across WT Mbd3 peaks plotted for ER-ChIP in uninduced cells (left) or after 48 hr of tamoxifen treatment (middle) and for Mbd3-ChIP in WT ESCs (right). Mean signal is plotted across the top.

(G) qRT-PCR using nascent RNA of indicated genes over 48 hr of the time course of tamoxifen addition (mean of relative expression ± SEM; plotted relative to time 0). Tamoxifen was added to either the *Mbd3* inducible line (in red) or to the parental, *Mbd3*-null line as a control (in black). N $\geq$ 3 biological replicates.

(H) Unsupervised clustering of nascent RNA-seq during the Mbd3 induction time course. Significant changes ($|FC| > 2$; $p < 0.05$) in transcript level compared to time 0 are shown by gray bars in the left-hand panels. Triplicate samples were prepared and sequenced for each time point. See also Figures S1, S2, and S3.

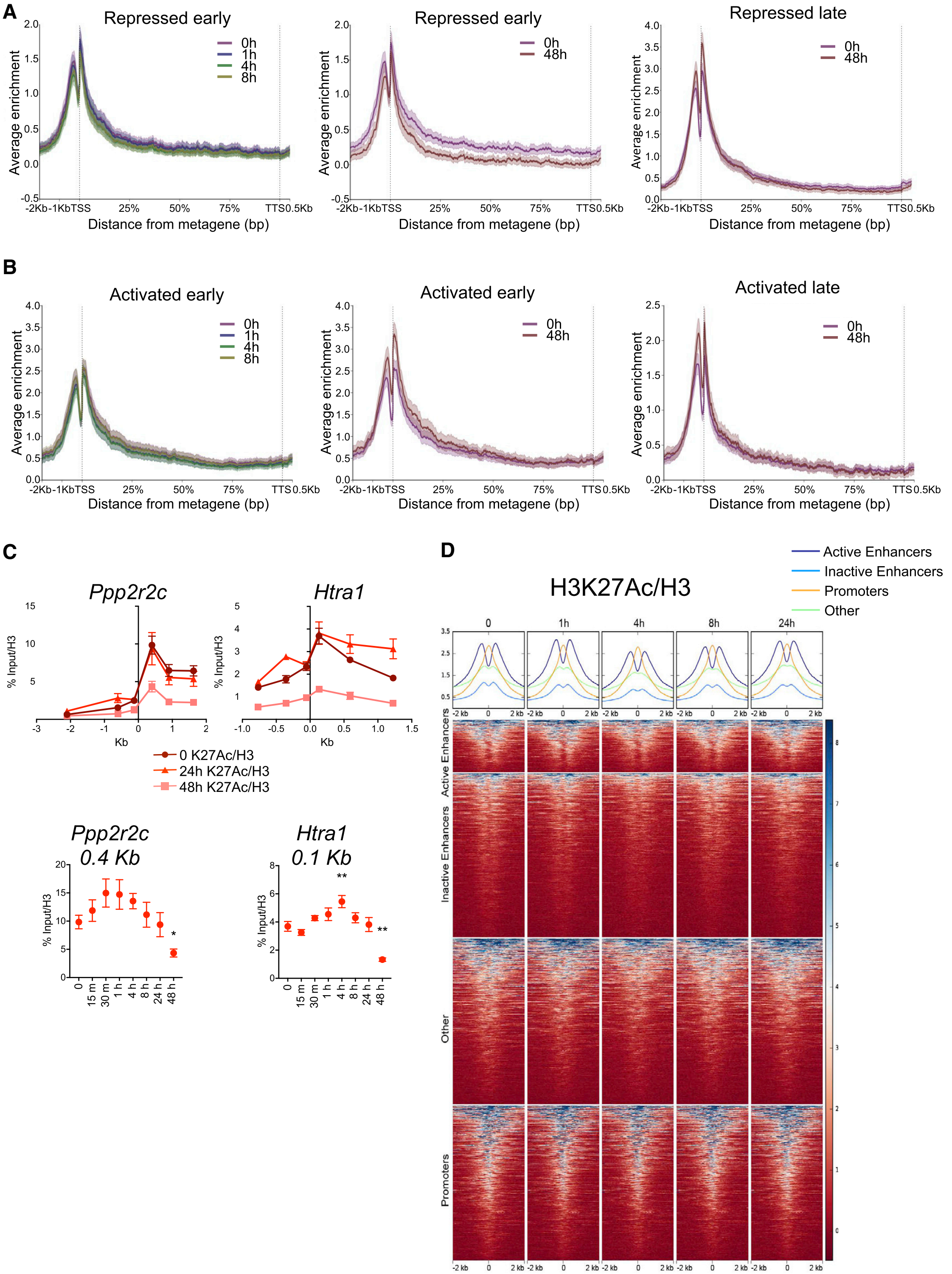

Figure 2. H3K27Ac Levels Do Not Immediately Correlate with NuRD-Induced Gene Expression Changes

(A) H3K27Ac ChIP-seq data normalized to H3 ChIP-seq at indicated time points of tamoxifen addition are plotted across a metagene for genes showing significant reduction in mRNA levels within 8 hr ("repressed early") or only after 24–48 hr ("repressed late"). Data are plotted as mean ± 95% confidence intervals (N = 3 biological replicates).

(B) As in (A) but for genes showing significant increases early (≤8 hr) or late (24–48 hr).

(legend continued on next page)

To determine whether this activity is used by the complex to drive transcriptional changes, we assessed how induction of NuRD impacted chromatin structure using micrococcal nuclease (MNase) digestion followed by high-throughput sequencing (MNase-seq). MNase cuts DNA efficiently at relatively open chromatin but digests less efficiently at sites associated with DNA binding proteins or nucleosomes and can therefore be used to map regions of open chromatin and to define sequences frequently associated with nucleosomes. Mbd3 induction resulted in a rapid (<30 min) and pronounced increase in protection from MNase digestion at Chd4- and Mbd3-bound sites genome-wide (Figure 3A). This manifested as an increase in MNase protection at sites of relatively open chromatin, such as enhancers and transcription start sites (Figure 3B). NuRD activity had no detectable influence over the spacing of positioned nucleosomes adjacent to CTCF-bound sites (Figure 3A), in contrast to reported effects for cells lacking the NuRF or SNF2H chromatin remodelers (Bohla et al., 2014; Kwon et al., 2016; Qiu et al., 2015; Wiechens et al., 2016). Considering that Chd4 targeting does not change significantly over this time course (Figure S2D), this pronounced Mbd3-induced change in chromatin structure may indicate that Mbd3 affects not only the stability of Chd4 association with chromatin but also its activity.

MNase-qPCR enabled us to determine more precisely how Mbd3/NuRD influenced chromatin structure at two specific genes responsive to NuRD activity: *Ppp2r2c* and *Bmp4* (Figure 1G). Positioned nucleosomes could be detected as regularly spaced peaks of MNase-resistant chromatin in *Mbd3*-null ESCs at enhancers and promoters associated with these genes (Figures 3C and 3D). Induction of Mbd3 activity imparted very little change in this structure at the *Ppp2r2c* promoter but resulted in pronounced changes at an enhancer located within intron 1 of the gene (Figure 3C). Specifically, DNA at position +8.6 kb in null cells rapidly lost MNase protection, which we interpret as a loss of a positioned nucleosome, and positions adjacent to those, at +8.3 kb (which coincides with the peak of Mbd3 binding) and +8.7 kb, gained protection with similar kinetics, consistent with increased nucleosome occupancy. Similarly, at the *Bmp4* promoter, a MNase-resistant site coinciding with the peak of Mbd3 binding (+0.12 Kb) was quickly lost, and MNase resistance was gained at flanking sites (−0.14 Kb and +0.31 Kb; Figure 3D). These precise changes in MNase sensitivity occurred rapidly (between 30 min and 1 hr) in response to NuRD induction and indicate active remodeling of nucleosome positioning within these regulatory elements. Mbd3-dependent changes to chromatin structure at these regulatory sequences were detectable prior to a detectable change in transcription of either gene (Figure 1G), consistent with induction, rather than response to a change in gene expression.

MNase sensitivity is usually interpreted in relation to nucleosome occupancy and positioning, but it is formally possible that some instances of nuclease protection could arise from NuRD occupancy, masking or exposing specific nucleosome-bound sites. If changes in MNase sensitivity are observed due to NuRD-mediated nucleosome movement, these should be dependent upon ATPase-dependent chromatin remodeling by Chd4 (Tong et al., 1998; Wade et al., 1998; Xue et al., 1998; Zhang et al., 1998). ESCs constitutively expressing an ATPase mutant Chd4 (Feng and Zhang, 2001) were not viable in long-term culture, so to test this, we expressed a doxycycline-inducible cDNA encoding either wild-type or an ATPase mutant Chd4 in Mbd3 inducible ESCs (Figure 4A). We reasoned that the over-expressed mutant protein would compete with endogenous Chd4 for binding at sites of action, where the ATPase mutant version would be unable to remodel nucleosomes and would thus impede any remodeling-dependent functions of NuRD. Chd4 expression was induced 18 hr prior to onset of the tamoxifen time course, which gave robust expression of both wild-type and mutant Chd4 (Figures 4A and 4B). We could detect both wild-type and ATPase mutant Chd4 associating with chromatin to similar extents, which did not interfere with the association of induced Mbd3 (Figure S4). When tamoxifen was added in the presence of the ATPase mutant Chd4, nucleosome movement at both the *Ppp2r2c* enhancer and *Bmp4* promoter was considerably slower than when tamoxifen was added either in the presence of wild-type Chd4 or in cells in which mutant Chd4 expression was not induced (Figure 4C). This also resulted in delayed transcriptional repression of both genes (Figure 4D). We therefore conclude that the observed changes in MNase protection and subsequent changes in transcription are dependent upon ATPase and nucleosome remodeling activity of Chd4 and are thus likely to result from NuRD-dependent chromatin remodeling.

NuRD Activity Alters the Protein Binding Repertoire of Regulatory Sequences

What effect might a change in chromatin structure have at sites of active transcription? Mbd3 and Chd4 binding patterns are highly correlated with those of pluripotency-associated transcription factors (Figure S1C; Miller et al., 2016; Stevens et al., 2017), so we hypothesized that NuRD may act generally to regulate transcription factor access to regulatory sequences. To test this hypothesis, we performed ChIP-seq for two important pluripotency-associated sequence-specific transcription factors (TFs), Klf4 and Nanog, and for a component of the kinase module of the Mediator complex, Med12, across the Mbd3 induction time course. No changes in the levels of nuclear Nanog, Klf4, or Med12 protein were observed across the time series (Figure S5A). Mbd3 induction resulted in an initial loss of Nanog enrichment at target sites globally, followed by an increase in enrichment through 24 hr (Figure 5A). Similarly, Klf4 enrichment was initially reduced at its targets upon Mbd3 induction, but levels were restored by 4 hr (Figure 5A). Global Med12

(C) ChIP-qPCR for H3K27Ac plotted relative to H3 ChIP across the *Ppp2r2c* and *Htra1* transcription start sites for 0, 24, and 48 hr following tamoxifen addition. Mean ± SEM; N ≥ 3 biological replicates. (Below) Data at the peak of enrichment from the panels above are displayed across the time course of tamoxifen exposure. Mean ± SEM (**p < 0.01 and *p < 0.05 relative to 0 hr). N = 2–5 biological replicates.

(D) ChIP-seq for H3K27Ac normalized to H3 ChIP for each time point of Mbd3 induction centered at peaks of NuRD binding ± 2 Kb, classified into active and inactive enhancers, promoters, or other sequences. Mean signal for each category is plotted across the top.

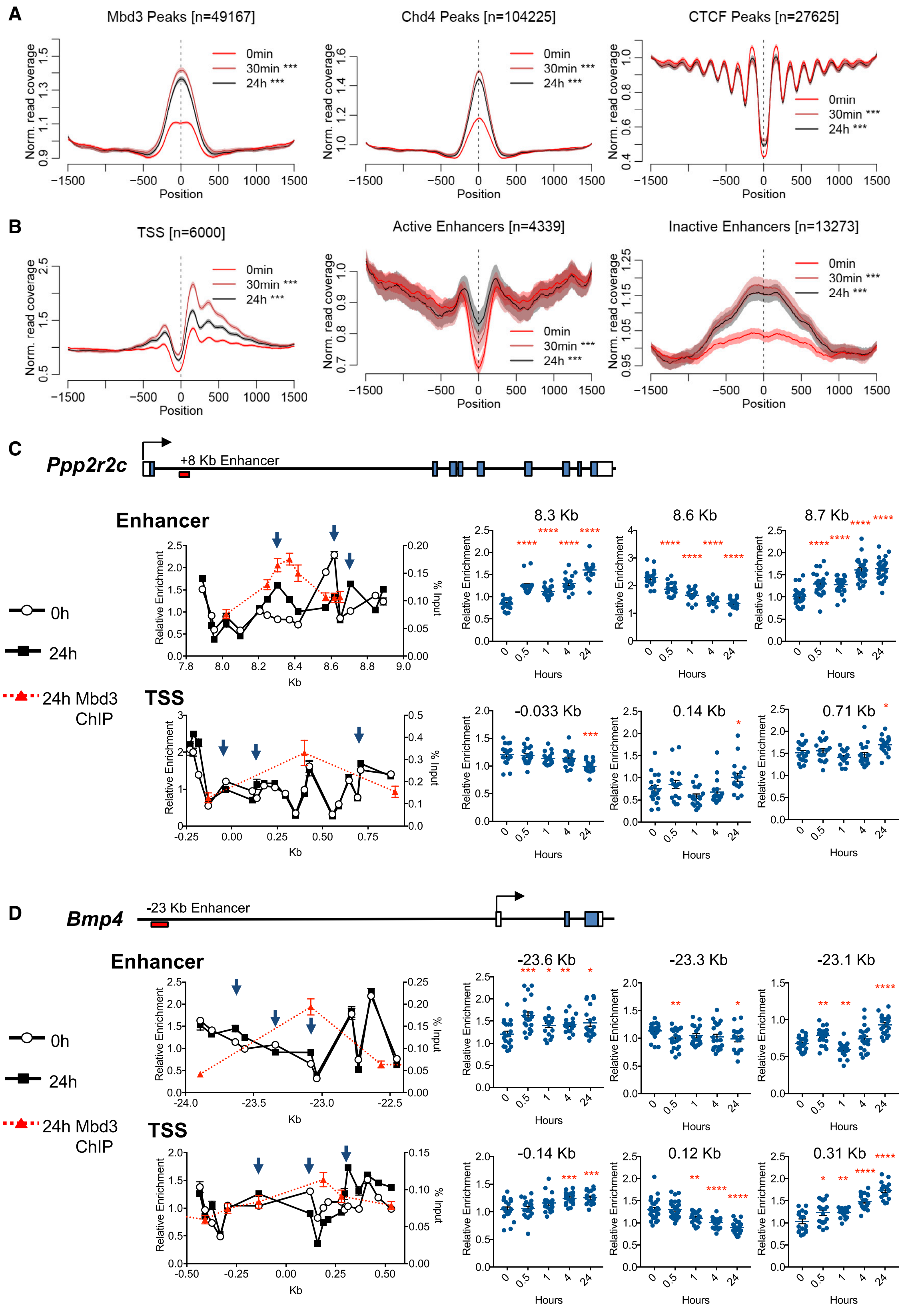

(legend on next page)

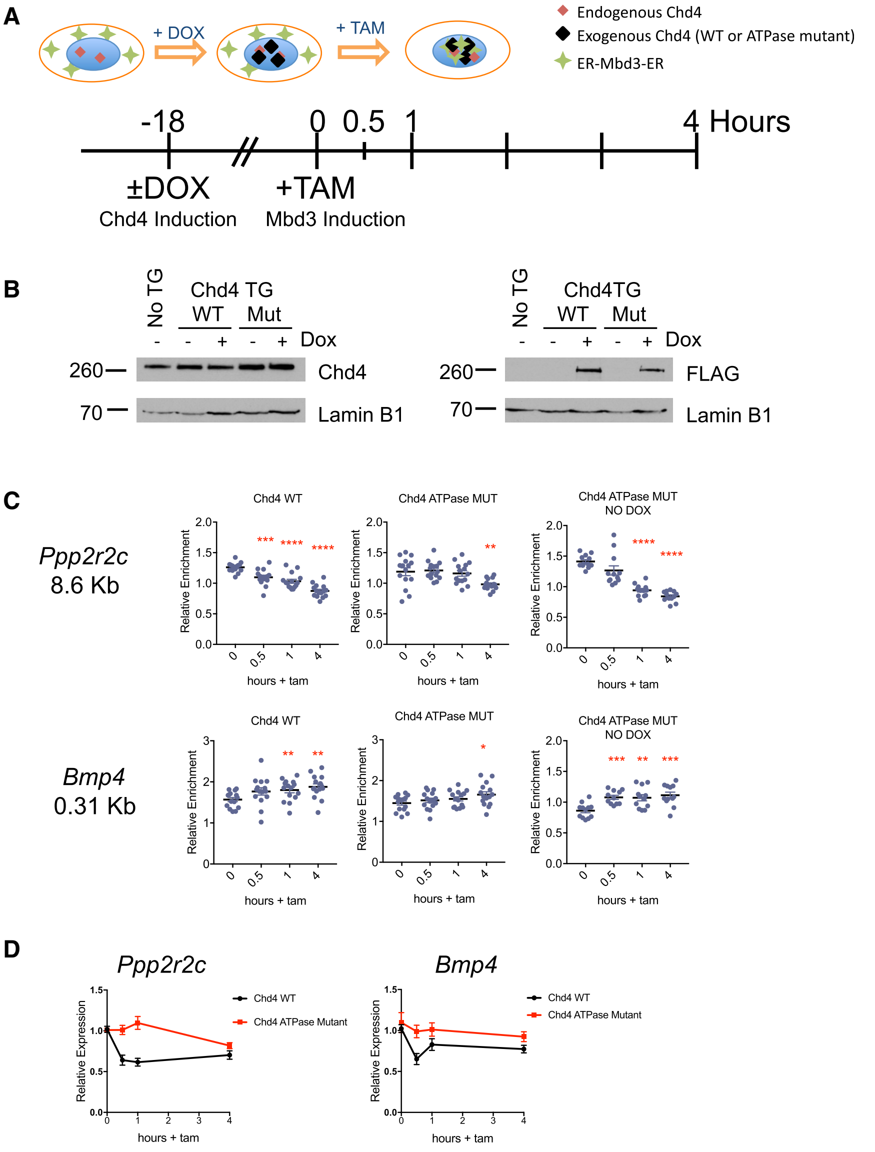

Figure 4. Nucleosome Remodeling Is Chd4 Dependent

(A) Timeline of the experiment. DOX was added to induce expression of the WT or mutant Chd4 cDNA 18 hr prior to tamoxifen addition, which induced nuclear translocation of Mbd3-ER.

(B) Nuclear extracts from ESCs expressing either the WT or ATPase mutant (Mut) Chd4 transgene (Chd4 TG) with and without DOX treatment were probed with an anti-Chd4 (left) or an anti-FLAG antibody (right). "No TG," ESCs with no Chd4-transgene. Lamin B1 serves as a loading control. Protein sizes are given at left in kilodaltons.

(C) MNase qPCR as in Figures 3C and 3D for indicated locations in the *Ppp2r2c* enhancer and *Bmp4* promoter in ESCs expressing the WT Chd4 transgene (left), the ATPase mutant Chd4 transgene (middle), and in cells in which expression of the ATPase mutant Chd4 was not induced with DOX (right). ****$p < 0.0001$, ***$p < 0.001$, **$p < 0.01$, and *$p < 0.05$ relative to 0 hr using a two-tailed t test. N = 4 or 5 biological replicates.

(D) Expression data of nascent RNA measured by qRT-PCR for *Ppp2r2c* or *Bmp4* across the tamoxifen induction time course in ESCs expressing the WT (Chd4 WT) or ATPase mutant (Chd4 ATPase mutant) CHD4 are plotted over time. N = 6–9 biological replicates. See also Figure S4.

enrichment remained stable during the first 4 hr of Mbd3 induction but increased significantly by 24 hr (Figure 5A). Thus, despite genome-wide reduction of chromatin accessibility at NuRD target sites (Figure 3A), this does not result in a global decrease in TF binding and indeed appears to enhance Nanog and Med12 binding to target sites in the longer term.

The above data indicate that NuRD does not regulate TF binding globally, but it remains possible that the complex can control TF binding at specific NuRD- and TF-binding loci. If NuRD-mediated chromatin remodeling directly regulates TF binding at a subset of target sites, we would expect to see a decrease in chromatin accessibility at sites that lose TF binding and vice versa. To this end, we first identified sites at which Nanog or Klf4 binding was significantly higher in wild-type cells ("higher in -WT") or in *Mbd3*-null cells ("higher in KO"). For Med12, we performed a similar analysis using *Mbd3*-null cells and inducible cells after 24 hr of induction. These are sites that we predict would gain or lose TF/Med12 binding, respectively, during Mbd3 induction. We then compared protein occupancy and MNase accessibility at these two different classes of sites for each protein across the time course. Sites with highest Nanog, Klf4, or Med12 binding in *Mbd3*-null cells all showed a significant loss of protein enrichment within 30 min of Mbd3 induction (Figure 5B, left panels). For all three proteins, this corresponded to an average increase in MNase protection across binding

Figure 3. NuRD Controls Nucleosome Density at Regulatory Sequences

(A and B) MNase-seq data collected after 0 or 30 min or 24 hr of tamoxifen treatment are plotted (A) across peaks of enrichment for indicated proteins defined in WT cells or (B) across indicated features (n = number of peaks/feature). Data are plotted as mean ± 95% confidence intervals. p values were calculated for each time point relative to time 0 for the center of the feature indicated with a dashed line. ***$p < 0.001$. N = 3 biological replicates.

(C and D) MNase-qPCR data (mean ± SEM) for the 0- and 24-hr time points plotted across an enhancer and the TSS for *Ppp2r2c* (C) and for *Bmp4* (D). The x axis indicates Kb relative to the annotated TSS for each gene. Overlaid in red are ChIP-qPCR data for Mbd3-ER at 24 hr post-tamoxifen addition. The blue arrows indicate the positions further analyzed in panels at right, where the horizontal line shows the mean. A schematic of each gene is shown above the ChIP-qPCR panels. Filled and open boxes represent coding and non-coding exons, respectively, and the red box below the line indicates the position of the relevant enhancer. N ≥ 6 biological replicates. ****$p < 0.0001$, ***$p < 0.001$, **$p < 0.01$, and *$p < 0.05$ relative to 0 hr using a two-tailed t test.

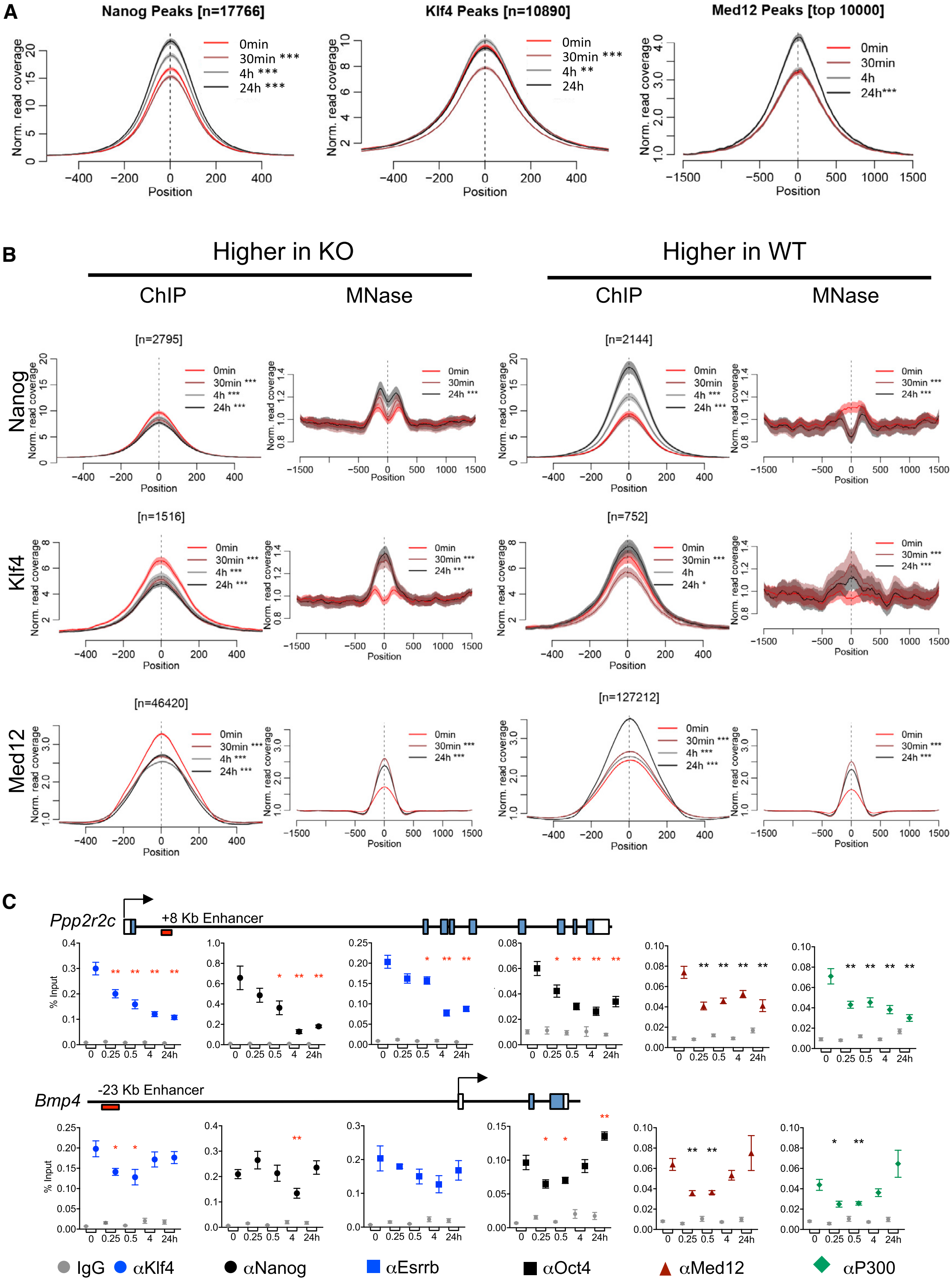

Figure 5. NuRD Controls Transcription Factor Access to Chromatin

(A) Protein binding at indicated time points across ChIP-seq peaks defined in WT cells (Nanog and Klf4) or at the most significant peaks identified at the 24-hr time point in the in the Mbd3 inducible cell line (Med12). Data are plotted as mean ± 95% confidence intervals. The later time points were compared with 0 hr, and significant differences in mean are indicated as **p < 0.01 or ***p < 0.001 using a two-tailed t test.

(legend continued on next page)

sites: for Nanog sites, this increase had begun by 30 min and continued to increase thereafter, although at Klf4 and Med12 sites, the greatest shift in MNase protection occurred within 30 min. These data are consistent with a model in which NuRD-mediated increases in nucleosome density interferes with or evicts Nanog, Klf4, and/or Med12 from this subset of bound sites.

Sites at which Nanog, Klf4, or Med12 binding was lower in Mbd3 KO cells than in WT cells (i.e., sites predicted to gain binding in response to NuRD activity) showed no consistent pattern of behavior. At Nanog sites, Mbd3 induction was associated with a rapid loss of MNase protection, consistent with NuRD acting to make these sites more accessible, although increases in Nanog protein binding were not detected until 4–24 hr (Figure 5B, right panels). At Klf4 and Med12 sites, induction of Mbd3 was accompanied by a rapid (30 min) increase in MNase protection. At Klf4 sites, this was first associated with a loss of Klf4 binding, consistent with the initial exclusion of Klf4 from these sites; however, subsequent restoration and gain of Klf4 enrichment occurred with no further change in chromatin accessibility (Figure 5B). At Med12 sites, the rapid increase in MNase protection did not appear to impact Med12 binding, and the predominant gain of Med12 enrichment between 4 and 24 hr was not associated with a large change in chromatin accessibility. Together, these data support a model in which NuRD-dependent chromatin remodeling can displace chromatin-bound proteins. Increased protein binding, however, is not tightly correlated to MNase protection and thus may be an indirect effect of NuRD activity.

To verify this model, we examined TF binding behavior in detail by ChIP-qPCR for Klf4, Nanog, Esrrb, Oct4, Med12, and P300 at two enhancers that both show changes in nucleosome structure during the NuRD induction time course (Figures 3C and 3D). The intronic *Ppp2r2c* enhancer showed an acute and sustained loss of all chromatin-associated proteins tested upon Mbd3 induction, coincident with rapid stabilization of the +8.3 Kb nucleosome (Figures 3C and 5C), resulting in an overall reduction in TF and coactivator binding at this site (Figure S5B). In contrast, at the upstream *Bmp4* enhancer, an initial loss of protein binding, consistent with a gain of MNase protection at the −23 Kb position, was followed by gradual recovery of protein binding levels over time (Figures 3C and 5D), resulting in either no change or a slight increase in protein biding by 24 hr (Figure S5C). We conclude that an acute NuRD-dependent increase in local nucleosome density can result in eviction of chromatin-bound proteins. Rather than creating inaccessible or "closed" chromatin, however, this initial clearance of proteins from regulatory sequences allows for a new protein-binding repertoire to be established. This resetting of regulatory element occupancy upon a now NuRD-defined nucleosome topology may result in altered recruitment and association of individual DNA-binding proteins.

NuRD Induction Displaces RNA Polymerase II from Transcription Start Sites

NuRD has a rapid and pronounced impact upon MNase sensitivity across transcription start sites globally (Figure 6A). Most notably, there is a large increase in MNase protection associated with the positioned nucleosomes immediately up- and downstream of transcriptional start sites (TSSs) at 30 min of Mbd3 induction. Although this increase is less pronounced by 24 hr, overall, there is a persistent rise in protection across TSSs globally relative to the uninduced state. This effect is seen irrespective of whether the associated genes show increased, decreased, or unchanged expression levels 48 hr after Mbd3 induction (Figure 6A).

Paused RNA polymerase II, which is found widely across transcription start sites in metazoan cells, is often associated with nucleosome depletion (Gilchrist et al., 2008). To address how NuRD-induced changes in nucleosome occupancy at TSSs correspond to the status of the transcription machinery, we performed ChIP-seq for the initiating form of RNA polymerase II (phosphorylated at the serine 5 position of the C-terminal repeat; S5P). Mbd3 induction resulted in a rapid ($\leq$30 min) decrease in the amount of S5P RNA polymerase II associated with transcription start sites globally (Figure 6B). This decrease continued through 4 hr, and then levels had increased again by 24 hr. This pattern was seen regardless of whether the associated genes showed altered transcription after Mbd3 induction (Figure 6B). Throughout the time course, no changes in global levels of S5 phosphorylated RNA polymerase II were detectable by immunoblotting (Figure S5A).

Mbd3-dependent increase in nucleosome density is most pronounced immediately downstream of the transcription start site. If this was the cause of RNA polymerase II displacement, we would expect to see a more pronounced loss of RNA polymerase II at the 5′ ends of genes than in gene bodies. To test this, we measured traveling ratios across the Mbd3 induction time course for all TSSs (Figure 6C). Traveling ratios give an indication of the relative abundance of RNA polymerase at promoters versus the gene body (Adelman and Lis, 2012). They can be used to indicate the degree of transcriptional pausing, although initiation rate and elongation rate also influence traveling ratio (Ehrensberger et al., 2013). Induction of NuRD activity resulted in a global decrease in traveling ratio within 30 min of Mbd3 induction, which reached a minimum at 4 hr (Figure 6D). This was true regardless of the direction of expression change (Figure S6). Together, these data indicate that a NuRD-directed increase in nucleosome occupancy at transcription start sites globally results in a decrease in TSS-associated RNA polymerase II occupancy.

It was curious that both the increase in nucleosome occupancy and the decrease in associated RNA polymerase II occurred irrespective of whether associated genes were repressed, activated,

(B) Protein binding or MNase protection are plotted across sites defined as having significantly increased binding in *Mbd3*-null ESCs versus WT cells (Nanog and Klf4) or were called as peaks at 0 hr, but not at 24 hr, in the Mbd3 induction time course (Med12; higher in KO) or the opposite (higher in WT) for each protein. The later time points were compared with 0 hr, and significant differences in mean are indicated as *$p < 0.05$ or ***$p < 0.001$ using a two-tailed t test.

(C) ChIP-qPCR for indicated proteins at the peak of binding for each feature (Figure S5B) at the indicated *Ppp2r2c* or *Bmp4* enhancers across the time course of tamoxifen exposure. A schematic of each gene is shown above the ChIP-qPCR panels. Time after tamoxifen addition in hours is indicated along the x axis. Mean ± SEM is plotted for all points. **$p < 0.01$ and *$p < 0.05$ relative to 0 hr using a two-tailed t test. See also Figure S5.

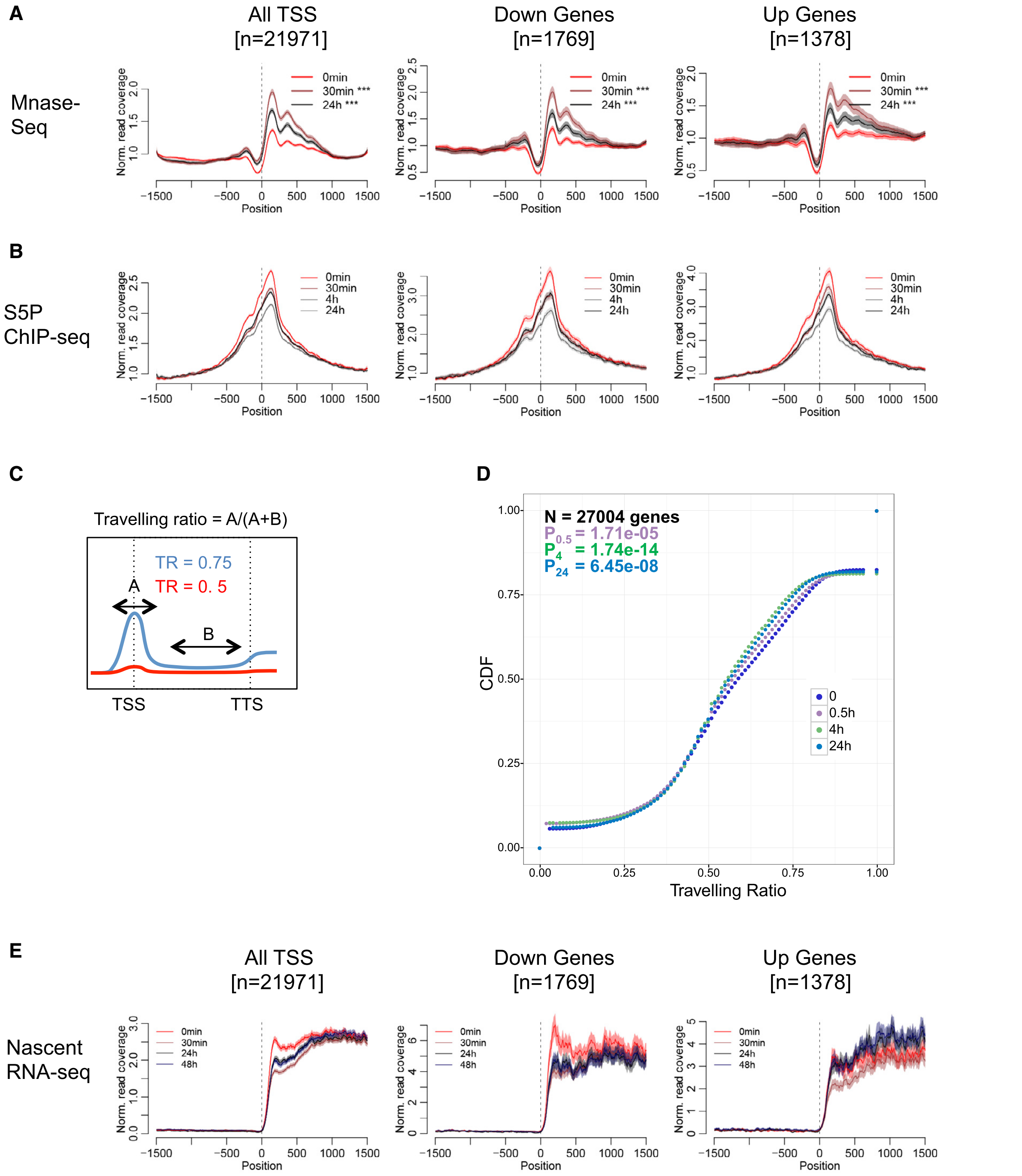

Figure 6. NuRD Induction Results in Loss of RNA Polymerase II from Transcription Start Sites and a Transient Reduction in Nascent RNAs

(A) MNase-seq reads plotted as in Figure 3A across all TSS or just those showing reduced (down genes) or increased (up genes) expression after 48 hr of tamoxifen treatment. p values were calculated as in Figure 3A. ***p < 0.001.

(B) ChIP-seq signal for RNA polymerase II (S5P) plotted as in (A).

(C) Schematic of traveling ratio calculation. The blue line represents S5P ChIP-seq signal across a paused gene (TR = 0.75), whereas the red line shows signal across an unpaused gene (TR = 0.5). TSS, transcription start site; TTS, "transcription termination site" (defined as polyA addition site).

(D) Cumulative traveling ratio calculated from RNA polymerase II (S5P) ChIP-seq data at time 0 (dark purple), 30 min (0.5H; light purple), 4 hr (green), and 24 hr (blue). CDF, cumulative density function. p values are given for each time point compared to time 0.

(E) Nascent RNA-seq plotted as in (A). Only data for the + strand are plotted as confidence intervals. N = 3 biological replicates. See also Figure S6.

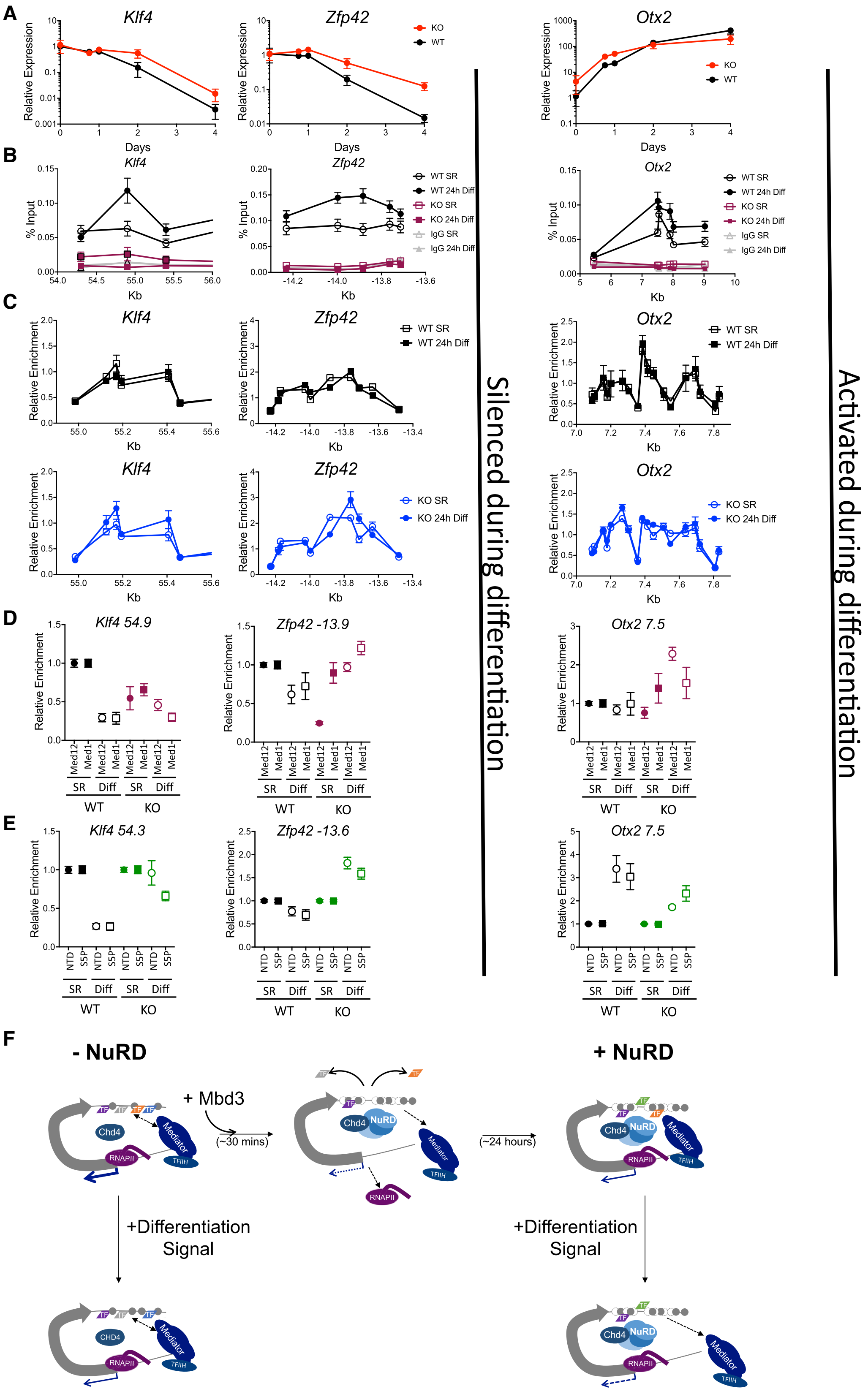

(legend on next page)

or unchanged during the Mbd3 induction time course. To investigate this in more detail, we monitored the change in nascent RNA-seq reads across transcription start sites. This showed a specific effect at transcripts associated with the very 5′ ends of genes, precisely where the largest changes in nucleosome and RNA polymerase II occupancy are observed. Specifically, induction of Mbd3 resulted in a sharp decrease in transcripts localizing within the first 750 bp of genes after 30 min, which then increased to a steady-state level by 24 hr (Figure 6E). Examining those genes specifically repressed after Mbd3 induction (down genes) revealed a similar decrease in transcripts at 30 min, and these remained reduced through 48 hr, consistent with a reduction of transcription at these genes by 48 hr. Genes activated by Mbd3 induction (up genes) again showed a significant decrease in nascent RNA mapping to 5′ ends of the genes at 30 min, but levels increased again by 24 and 48 hr.

Thus the initial response to acute Mbd3 induction is the same for all genes: increase in nucleosome density, loss of RNA polymerase II, and reduction in transcriptional output associated with 5′ ends. At a subset of genes, re-establishment of RNA polymerase II levels results in a stable reduction in overall transcription (down genes), although at other genes, the result of re-establishment is an overall increase in stable transcript levels (up genes). Therefore, by resetting the local nucleosome landscape and clearing the TSS of RNA polymerase II so that a new transcriptional state is established, one chromatin remodeling complex can act to repress some genes and activate others. The activity exerted by NuRD is the same at all target sites, namely altering nucleosome density. Yet the impact this has differs at specific genes, presumably due to variation in the architecture of the regulatory element in question.

NuRD Controls Nucleosome Structure and Enhancer Access during an ESC State Transition

NuRD facilitates exit from the self-renewing state in ESCs by modulating gene expression levels (Reynolds et al., 2012a). We asked whether the NuRD-dependent control of chromatin structure and protein binding identified in our inducible system also occurred at NuRD target genes during exit from the ESC state. ESCs cultured in self-renewal conditions express naive pluripotency markers, such as *Klf4* and *Zpf42* (*Rex1*), but not the primed pluripotency marker *Otx2*. Upon withdrawal of self-renewal factors, cells exit naive pluripotency, downregulating *Klf4* and *Zfp42* and upregulating *Otx2.* These initial effects are observed in both WT and *Mbd3*-null cells, but in the absence of Mbd3, gene expression changes proceed more slowly (Figure 7A). These kinetic differences enabled us to explore the mechanisms underlying transcriptional regulation in the presence or absence of endogenous Mbd3 as cells underwent a cell state transition. In contrast to our inducible system that illustrates the mechanistic effects of acute NuRD recruitment to sites of action, this allows us to monitor in detail the molecular events occurring at a set of regulatory regions involved in developmentally relevant transcriptional changes. We focused on changes at three enhancers after 24 hr in differentiation conditions, as this is the point immediately preceding a difference in the kinetics of silencing of *Klf4* and *Zfp42* and activation of *Otx2* between WT and *Mbd3*-null ESCs (Figure 7A).

Enhancers associated with *Klf4*, *Zfp42*, and *Otx2* were bound by Mbd3 in self-renewing conditions, though Mbd3 enrichment increased after 24 hr in differentiation conditions (Figure 7B). Induction of differentiation in WT cells resulted in little overall change in the nucleosome structure at any of the enhancers analyzed after 24 hr (Figure 7C). In self-renewing *Mbd3*$^{-/-}$ cells, there were small but distinct differences to the nucleosome patterns of the *Zfp42* and *Otx2* enhancers relative to those seen in WT cells (Figure 7C). Induction of differentiation in *Mbd3*-null ESCs resulted in substantial changes to the MNase protection pattern of all enhancers analyzed, indicating that NuRD normally acts to stabilize the nucleosome structure of these enhancers during a change in transcription (Figure 7C).

During differentiation-induced transcriptional changes in WT cells, components of the Mediator complex, Med12 and Med1, showed reduced enrichment at *Klf4* and *Zfp42* enhancers but remained stable at the *Otx2* enhancer (Figure 7D). In the absence of NuRD, Med12 levels were reduced at the enhancers in self-renewing conditions consistent with our ChIP-seq data (Figure 6A). *Mbd3*-null cells maintained in 2iL conditions are under strict selection to maintain appropriate expression levels of genes in the pluripotency regulatory network, and the reduced association of Mediator components with these enhancers may be part of how cells adapt to not having NuRD activity. Unlike in WT cells, though,

Figure 7. Inappropriate Enhancer Chromatin Remodeling and Protein Binding in *Mbd3*-Null ESCs during Lineage Commitment

(A) Expression of *Klf4*, *Zfp42*, and *Otx2* over a differentiation time course relative to that in WT cells in 2iL conditions. Mean ± SEM; N $\geq$ 3 biological replicates.

(B) ChIP-qPCR for Mbd3-FLAG in WT (black) and *Mbd3*-null (magenta) ESCs across indicated enhancer sequences in 2iL (self-renewing conditions [SR]) or after 24 hr in differentiation conditions (24h Diff). Mean ± SEM; N $\geq$ 4 biological replicates.

(C) MNase-qPCR profiles across enhancers associated with *Klf4*, *Zfp42*, and *Otx2* are plotted for WT (black) or *Mbd3*-null ESCs (blue) in SR and after 24h Diff. Mean ± SEM; N $\geq$ 3 biological replicates.

(D) ChIP-qPCR for Med12 and Med1 in WT (black) or *Mbd3*-null ESCs (magenta) before and after 24 hr in differentiation media. Data are plotted relative to levels in WT at time 0. Mean ± SEM; N $\geq$ 3 biological replicates.

(E) ChIP-qPCR for either total (NTD) or serine 5 phosphorylated (S5P) RNA polymerase II as in (D). Data are plotted relative to time 0. Mean ± SEM; N $\geq$ 3 biological replicates.

(F) Model of how NuRD controls transcription. In the absence of Mbd3 (top left), NuRD does not form and regulatory sequences for a hypothetical gene adopt a specific nucleosome structure (gray spheres), are bound by transcription factors (TFs), and associate loosely with Mediator and with RNA polymerase II at the start of a gene (gray arrow). Adding back Mbd3 results in rapid NuRD reformation, an increase in nucleosome density, and eviction of TFs, Mediator, and RNA polymerase I from the regulatory sequences in the first 30 min (top middle). By 24 hr, the NuRD-dictated nucleosome structure has adopted a new transcription factor repertoire and is more stably associated with Mediator. This is the WT situation (top right). When cells are induced to differentiate, NuRD holds the nucleosome structure in place while Mediator is excluded in preparation for a change in transcriptional output (bottom right). In the absence of Mbd3 (bottom left), the differentiation signal results in a shift in nucleosome structure that does not exclude Mediator, making it less likely that the required change in transcription will occur.

no further reduction in Mediator component enrichment was seen upon induction of differentiation in the absence of Mbd3, and at the *Zfp42* and *Otx2* enhancers, Med12 enrichment actually increased relative to the undifferentiated state (Figure 7D).

Consistent with the observed changes in transcriptional output of each gene in WT cells, induction of differentiation also resulted in reduced association between the *Klf4* or *Zfp42* enhancers with RNA polymerase II and with increased association at the *Otx2* enhancer (Figure 7E). This change in enhancer-RNA polymerase II interaction in *Mbd3*$^{-/-}$ cells was altered at enhancers corresponding to all three genes. At the *Klf4* and *Zfp42* enhancers, the interaction was abnormally high upon differentiation, consistent with a defect in silencing, and at *Otx2*, the degree of interaction did not increase as much as in WT cells, consistent with impaired gene activation.

Together, these data show that NuRD activity is required to maintain an appropriate nucleosome structure and protein binding repertoire at three different regulatory sequences when the associated genes are undergoing a change in expression status: in this case, as a result of a developmental transition. Assuming that this holds true more generally across the genome, we propose a model in which appropriate transcriptional response to a given stimulus requires NuRD-dependent control of local nucleosome structure and that dictates the ability of different proteins to stably associate with that sequence (Figure 7F). By controlling the nucleosome structure of regulatory sequences, NuRD acts at enhancers and promoters to regulate transcriptional programs. We suggest that it is this fine-tuning of enhancer responsiveness and transcriptional output on a genome-wide scale that is required for a pluripotent cell to properly orchestrate a lineage commitment event.

DISCUSSION

Although many chromatin remodeling proteins are essential for mammalian development, the actual mechanics of how they influence transcription remain ill defined. In particular, the NuRD complex has been something of a puzzle: although this complex was originally defined as a co-repressor, it is present at all sites of active transcription in ESCs and serves to both activate and repress transcription of a similar number of genes. Here, we use an inducible system with fine temporal resolution to resolve this apparent paradox. Induction of NuRD activity results in rapid reorganization of nucleosome structure at enhancers and promoters genome-wide, clearing some chromatin-bound proteins and RNA polymerase II from these sites. This initial eviction of chromatin-bound proteins is followed by re-establishment of the protein binding repertoire, which may then differ from the original configuration (model, Figure 7F). At most genes, this resetting of enhancers and promoters has only a transient impact on nascent RNA production at transcription start sites. At a subset of genes, however, this revised protein binding landscape results in altered mRNA output, with similar numbers of genes showing increased or decreased expression. We further show that NuRD-mediated control of nucleosome structure at enhancers of three developmentally relevant target genes occurs during transcriptional responses during a developmental transition.

NuRD's broad distribution across all sites of active transcription in ESCs is consistent with recruitment arising from an affinity for open chromatin or the presence of some component of the RNA polymerase machinery. Whereas it is clear that, in some cases, transcription factors can recruit NuRD to chromatin, Mbd3 enrichment is quickly lost at transcribed genes upon addition of transcription inhibitors (data not shown), supporting targeting mechanisms more tightly linked to the transcription process itself. The downstream effect of nucleosome remodeling activity differs in a locus-specific manner. What governs whether a gene is up- or downregulated in response to NuRD remains to be defined but is likely to be determined by the underlying DNA sequence and affinity of transcription factors for sites therein.

All of the actively transcribed genes in ESCs could be deemed "direct NuRD targets" simply by virtue of having Mbd3/Chd4 ChIP peaks at their promoters (Figure S1). Using standard practice, each of the ~2,000 genes that show differential expression between *Mbd3* WT and *Mbd3* KO ESCs could therefore be argued to be "directly regulated by NuRD". This does not distinguish between gene expression changes occurring as a primary consequence of Mbd3 loss and those that occurred as a consequence of the cells having been selected to survive and proliferate in the absence of Mbd3/NuRD. Differentiating between these two scenarios is important for understanding how NuRD, or indeed any chromatin remodeler, regulates transcription. Furthermore, combining ChIP-seq data with observations from steady state knockout lines led us to propose that NuRD can act as both activator and repressor of transcription (Reynolds et al., 2013); however, indirect effects could also be responsible for this observation. Using our inducible system, we are able to determine direct consequences of NuRD activity, clearly defining a chain of events starting with reformation of NuRD and ending with stable changes in transcription. This not only confirms that NuRD is capable of both increasing and decreasing transcription but also provides a model for how one remodeling complex can influence transcription in opposing directions at distinct loci.

The *in vivo* targets of the lysine deacetylase activity of NuRD have not been defined, although anti-correlation of H3K27Ac levels with NuRD function has been observed in steady-state conditions (Reynolds et al., 2012b). *In vitro*, the histone deacetylase components of the NuRD complex show little substrate specificity (Zhang et al., 2016). In our system, changes in levels of H3K27Ac are not associated with early stages of transcriptional regulation but rather follow gene expression changes (Figure 2). This result is consistent with studies in yeast and flies, which found that transcription induction in some contexts does not require covalent histone modifications (Henikoff and Shilatifard, 2011; Pérez-Lluch et al., 2015; Zhang et al., 2014). A recent study found that histone deacetylase activity was dispensable for NuRD-dependent silencing of Ikaros target genes in pre-B cells but rather served to reinforce silencing (Liang et al., 2017). The synergistic interaction between NuRD and PRC2 is most consistent with this maintenance role for NuRD-associated deacetylase activity.

In a cellular context, chromatin remodelers do not act in isolation. The pattern of NuRD binding to chromatin closely resembles that of the activating chromatin remodeling complex BAF (Ho et al., 2011; King and Klose, 2017). Although NuRD acts to

increase nucleosome density (Figure 3), BAF has the opposite effect, acting to remove nucleosomes from regulatory sequences (de Dieuleveult et al., 2016; Miller et al., 2017; Morris et al., 2014). Why should these two opposing chromatin remodeling activities be present at the same regulatory sequences in ESCs? If the two complexes are indeed acting in direct opposition at the same sites but their activities are closely matched, then their opposing activities could serve to finely tune the activity levels of regulatory sequences. A balance of nucleosome remodeling activity at enhancers could similarly provide fine tuning of enhancer activity but also serve to make them particularly responsive to inductive (or repressive) signals. For example, an enhancer where BAF activity dominates may have low nucleosome density and be easily accessible to binding by an inductive TF. If the activity of NuRD dominated at that same enhancer, then the nucleosome density would be higher, resulting in a less accessible enhancer, which might require a higher concentration of TF for induction. This is consistent with our findings that absence of NuRD does not prevent signal-induced changes in gene expression but rather changes the kinetics and/or magnitude of the transcriptional changes (Figure 7; Reynolds et al., 2012a). Such fine-tuning of promoter or enhancer chromatin may well have no overall impact on many genes, for which a level of NuRD- or BAF-modulated enhancer tweaking has no influence on transcriptional output. This is consistent with the observations that loss of either NuRD or BAF activity has no impact on the transcript levels of the vast majority of genes in ESCs (Figure S1E; Ho et al., 2011; Stevens et al., 2017). In a developmental context, such as during ESC differentiation, where cells switch from one transcriptional program to another, this balance between remodeling activities at regulatory elements is critical.

STAR★METHODS

Detailed methods are provided in the online version of this paper and include the following:

- KEY RESOURCES TABLE
- CONTACT FOR REAGENT AND RESOURCE SHARING
- EXPERIMENTAL MODEL AND SUBJECT DETAILS
  - Tissue culture
- METHOD DETAILS
  - Immunoprecipitation and western blotting
  - ChIP, ChIP-seq and ChIP-seq data analyses
  - RNA-seq and expression analysis
  - MNase-seq and MNase qPCR
  - Aggregate profiles of MNase-seq, Nascent RNA-seq and ChIP-seq
- QUANTIFICATION AND STATISTICAL ANALYSIS
- DATA AND SOFTWARE AVAILABILITY

SUPPLEMENTAL INFORMATION

Supplemental Information includes six figures and three tables and can be found with this article online at https://doi.org/10.1016/j.molcel.2018.06.003.

A video abstract is available at https://doi.org/10.1016/j.molcel.2018.06.003#mmc5.

ACKNOWLEDGMENTS

We thank R. Klose, A. Smith, E. Laue, K. Helin, and BDH Group members for useful discussions. We also thank R. Bandiera, M. Paramor, S. Lees, and A. Hendrich for technical advice and support. M.X. was funded through an EMBL PhD studentship, E.J. through a BBSRC PhD studentship, and J.S. through an MRC PhD studentship. Funding in the B.H. lab was provided by a Wellcome Trust Senior Fellowship (098021/Z/11/Z) and the EU FP7 Integrated Project “4DCellFate” (277899). Funding in the P.B. lab was provided by EMBL and the BBSRC (BB/M004023/1). B.H. and P.B. labs further benefit from core funding to the Cambridge Stem Cell Institute from the Wellcome Trust and Medical Research Council (079249/Z/06/I).

AUTHOR CONTRIBUTIONS

The study was conceived and designed by N.R., M.X., and B.H. N.R., S.G., J.S., R.F., and B.H. generated the data; S.B., M.X., E.J., M.R., R.L., S.D., and P.B. analyzed sequencing data; B.H., P.B., and S.D. oversaw the project; and B.H. wrote the paper with input from all authors.

DECLARATION OF INTERESTS

The authors declare no competing interests.

Received: April 19, 2017
Revised: March 20, 2018
Accepted: May 31, 2018
Published: June 28, 2018

REFERENCES

Adelman, K., and Lis, J.T. (2012). Promoter-proximal pausing of RNA polymerase II: emerging roles in metazoans. Nat. Rev. Genet. *13*, 720–731.

Aguilera, C., Nakagawa, K., Sancho, R., Chakraborty, A., Hendrich, B., and Behrens, A. (2011). c-Jun N-terminal phosphorylation antagonises recruitment of the Mbd3/NuRD repressor complex. Nature *469*, 231–235.

Allen, H.F., Wade, P.A., and Kutateladze, T.G. (2013). The NuRD architecture. Cell. Mol. Life Sci. *70*, 3513–3524.

Bohla, D., Herold, M., Panzer, I., Buxa, M.K., Ali, T., Demmers, J., Krüger, M., Scharfe, M., Jarek, M., Bartkuhn, M., and Renkawitz, R. (2014). A functional insulator screen identifies NURF and dREAM components to be required for enhancer-blocking. PLoS ONE *9*, e107765.

de Dieuleveult, M., Yen, K., Hmitou, I., Depaux, A., Boussouar, F., Bou Dargham, D., Jounier, S., Humbertclaude, H., Ribierre, F., Baulard, C., et al. (2016). Genome-wide nucleosome specificity and function of chromatin remodellers in ES cells. Nature *530*, 113–116.

Ehrensberger, A.H., Kelly, G.P., and Svejstrup, J.Q. (2013). Mechanistic interpretation of promoter-proximal peaks and RNAPII density maps. Cell *154*, 713–715.

ENCODE Project Consortium (2012). An integrated encyclopedia of DNA elements in the human genome. Nature *489*, 57–74.

Feng, Q., and Zhang, Y. (2001). The MeCP1 complex represses transcription through preferential binding, remodeling, and deacetylating methylated nucleosomes. Genes Dev. *15*, 827–832.

Gilchrist, D.A., Nechaev, S., Lee, C., Ghosh, S.K., Collins, J.B., Li, L., Gilmour, D.S., and Adelman, K. (2008). NELF-mediated stalling of Pol II can enhance gene expression by blocking promoter-proximal nucleosome assembly. Genes Dev. *22*, 1921–1933.

Günther, K., Rust, M., Leers, J., Boettger, T., Scharfe, M., Jarek, M., Bartkuhn, M., and Renkawitz, R. (2013). Differential roles for MBD2 and MBD3 at methylated CpG islands, active promoters and binding to exon sequences. Nucleic Acids Res. *41*, 3010–3021.

Hendrich, B., and Bird, A. (1998). Identification and characterization of a family of mammalian methyl-CpG binding proteins. Mol. Cell. Biol. *18*, 6538–6547.

Hendrich, B., Guy, J., Ramsahoye, B., Wilson, V.A., and Bird, A. (2001). Closely related proteins MBD2 and MBD3 play distinctive but interacting roles in mouse development. Genes Dev. *15*, 710–723.

Henikoff, S., and Shilatifard, A. (2011). Histone modification: cause or cog? Trends Genet. *27*, 389–396.

Ho, L., Miller, E.L., Ronan, J.L., Ho, W.Q., Jothi, R., and Crabtree, G.R. (2011). esBAF facilitates pluripotency by conditioning the genome for LIF/STAT3 signalling and by regulating polycomb function. Nat. Cell Biol. *13*, 903–913.

Hong, W., Nakazawa, M., Chen, Y.Y., Kori, R., Vakoc, C.R., Rakowski, C., and Blobel, G.A. (2005). FOG-1 recruits the NuRD repressor complex to mediate transcriptional repression by GATA-1. EMBO J. *24*, 2367–2378.

Kaji, K., Caballero, I.M., MacLeod, R., Nichols, J., Wilson, V.A., and Hendrich, B. (2006). The NuRD component Mbd3 is required for pluripotency of embryonic stem cells. Nat. Cell Biol. *8*, 285–292.

King, H.W., and Klose, R.J. (2017). The pioneer factor OCT4 requires the chromatin remodeller BRG1 to support gene regulatory element function in mouse embryonic stem cells. eLife *6*, e22631.

Kloet, S.L., Baymaz, H.I., Makowski, M., Groenewold, V., Jansen, P.W., Berendsen, M., Niazi, H., Kops, G.J., and Vermeulen, M. (2015). Towards elucidating the stability, dynamics and architecture of the nucleosome remodeling and deacetylase complex by using quantitative interaction proteomics. FEBS J. *282*, 1774–1785.

Kwon, S.Y., Grisan, V., Jang, B., Herbert, J., and Badenhorst, P. (2016). Genome-wide mapping targets of the metazoan chromatin remodeling factor NURF reveals nucleosome remodeling at enhancers, core promoters and gene insulators. PLoS Genet. *12*, e1005969.

Langmead, B., Trapnell, C., Pop, M., and Salzberg, S.L. (2009). Ultrafast and memory-efficient alignment of short DNA sequences to the human genome. Genome Biol. *10*, R25.

Le Guezennec, X., Vermeulen, M., Brinkman, A.B., Hoeijmakers, W.A., Cohen, A., Lasonder, E., and Stunnenberg, H.G. (2006). MBD2/NuRD and MBD3/NuRD, two distinct complexes with different biochemical and functional properties. Mol. Cell. Biol. *26*, 843–851.

Liang, Z., Brown, K.E., Carroll, T., Taylor, B., Vidal, I.F., Hendrich, B., Rueda, D., Fisher, A.G., and Merkenschlager, M. (2017). A high-resolution map of transcriptional repression. eLife *6*, e22767.

Liu, T. (2014). Use model-based analysis of ChIP-seq (MACS) to analyze short reads generated by sequencing protein-DNA interactions in embryonic stem cells. Methods Mol. Biol. *1150*, 81–95.

Love, M.I., Huber, W., and Anders, S. (2014). Moderated estimation of fold change and dispersion for RNA-seq data with DESeq2. Genome Biol. *15*, 550.

Low, J.K., Webb, S.R., Silva, A.P., Saathoff, H., Ryan, D.P., Torrado, M., Brofelth, M., Parker, B.L., Shepherd, N.E., and Mackay, J.P. (2016). CHD4 is a peripheral component of the nucleosome remodeling and deacetylase complex. J. Biol. Chem. *291*, 15853–15866.

Miller, A., Ralser, M., Kloet, S.L., Loos, R., Nishinakamura, R., Bertone, P., Vermeulen, M., and Hendrich, B. (2016). Sall4 controls differentiation of pluripotent cells independently of the nucleosome remodelling and deacetylation (NuRD) complex. Development *143*, 3074–3084.

Miller, E.L., Hargreaves, D.C., Kadoch, C., Chang, C.Y., Calarco, J.P., Hodges, C., Buenrostro, J.D., Cui, K., Greenleaf, W.J., Zhao, K., and Crabtree, G.R. (2017). TOP2 synergizes with BAF chromatin remodeling for both resolution and formation of facultative heterochromatin. Nat. Struct. Mol. Biol. *24*, 344–352.

Mohd-Sarip, A., Teeuwssen, M., Bot, A.G., De Herdt, M.J., Willems, S.M., Baatenburg de Jong, R.J., Looijenga, L.H.J., Zatreanu, D., Bezstarosti, K., van Riet, J., et al. (2017). DOC1-dependent recruitment of NURD reveals antagonism with SWI/SNF during epithelial-mesenchymal transition in oral cancer cells. Cell Rep. *20*, 61–75.

Morris, S.A., Baek, S., Sung, M.H., John, S., Wiench, M., Johnson, T.A., Schiltz, R.L., and Hager, G.L. (2014). Overlapping chromatin-remodeling systems collaborate genome wide at dynamic chromatin transitions. Nat. Struct. Mol. Biol. *21*, 73–81.

Moshkin, Y.M., Chalkley, G.E., Kan, T.W., Reddy, B.A., Ozgur, Z., van Ijcken, W.F., Dekkers, D.H., Demmers, J.A., Travers, A.A., and Verrijzer, C.P. (2012). Remodelers organize cellular chromatin by counteracting intrinsic histone-DNA sequence preferences in a class-specific manner. Mol. Cell. Biol. *32*, 675–688.

O'Shaughnessy, A., and Hendrich, B. (2013). CHD4 in the DNA-damage response and cell cycle progression: not so NuRDy now. Biochem. Soc. Trans. *41*, 777–782.

O'Shaughnessy-Kirwan, A., Signolet, J., Costello, I., Gharbi, S., and Hendrich, B. (2015). Constraint of gene expression by the chromatin remodelling protein CHD4 facilitates lineage specification. Development *142*, 2586–2597.

Pérez-Lluch, S., Blanco, E., Tilgner, H., Curado, J., Ruiz-Romero, M., Corominas, M., and Guigó, R. (2015). Absence of canonical marks of active chromatin in developmentally regulated genes. Nat. Genet. *47*, 1158–1167.

Qiu, Z., Song, C., Malakouti, N., Murray, D., Hariz, A., Zimmerman, M., Gygax, D., Alhazmi, A., and Landry, J.W. (2015). Functional interactions between NURF and Ctcf regulate gene expression. Mol. Cell. Biol. *35*, 224–237.

Rädle, B., Rutkowski, A.J., Ruzsics, Z., Friedel, C.C., Koszinowski, U.H., and Dölken, L. (2013). Metabolic labeling of newly transcribed RNA for high resolution gene expression profiling of RNA synthesis, processing and decay in cell culture. J. Vis. Exp. (78), 50195.

Ramírez, F., Ryan, D.P., Grüning, B., Bhardwaj, V., Kilpert, F., Richter, A.S., Heyne, S., Dündar, F., and Manke, T. (2016). deepTools2: a next generation web server for deep-sequencing data analysis. Nucleic Acids Res. *44* (W1), W160–W165.

Reynolds, N., Latos, P., Hynes-Allen, A., Loos, R., Leaford, D., O'Shaughnessy, A., Mosaku, O., Signolet, J., Brennecke, P., Kalkan, T., et al. (2012a). NuRD suppresses pluripotency gene expression to promote transcriptional heterogeneity and lineage commitment. Cell Stem Cell *10*, 583–594.

Reynolds, N., Salmon-Divon, M., Dvinge, H., Hynes-Allen, A., Balasooriya, G., Leaford, D., Behrens, A., Bertone, P., and Hendrich, B. (2012b). NuRD-mediated deacetylation of H3K27 facilitates recruitment of Polycomb Repressive Complex 2 to direct gene repression. EMBO J. *31*, 593–605.

Reynolds, N., O'Shaughnessy, A., and Hendrich, B. (2013). Transcriptional repressors: multifaceted regulators of gene expression. Development *140*, 505–512.

Saito, M., and Ishikawa, F. (2002). The mCpG-binding domain of human MBD3 does not bind to mCpG but interacts with NuRD/Mi2 components HDAC1 and MTA2. J. Biol. Chem. *277*, 35434–35439.

Salmon-Divon, M., Dvinge, H., Tammoja, K., and Bertone, P. (2010). PeakAnalyzer: genome-wide annotation of chromatin binding and modification loci. BMC Bioinformatics *11*, 415.

Sánchez-Díaz, A., Blanco, M.A., Jones, N., and Moreno, S. (2001). HBP2: a new mammalian protein that complements the fission yeast MBF transcription complex. Curr. Genet. *40*, 110–118.

Shimbo, T., Du, Y., Grimm, S.A., Dhasarathy, A., Mav, D., Shah, R.R., Shi, H., and Wade, P.A. (2013). MBD3 localizes at promoters, gene bodies and enhancers of active genes. PLoS Genet. *9*, e1004028.

Signolet, J., and Hendrich, B. (2015). The function of chromatin modifiers in lineage commitment and cell fate specification. FEBS J. *282*, 1692–1702.

Stark, R., and Brown, G. (2017). DiffBind: differential binding analysis of ChIP-seq peak data. http://bioconductor.org/packages/release/bioc/vignettes/DiffBind/inst/doc/DiffBind.pdf.

Stevens, T.J., Lando, D., Basu, S., Atkinson, L.P., Cao, Y., Lee, S.F., Leeb, M., Wohlfahrt, K.J., Boucher, W., O'Shaughnessy-Kirwan, A., et al. (2017). 3D structures of individual mammalian genomes studied by single-cell Hi-C. Nature *544*, 59–64.

Tong, J.K., Hassig, C.A., Schnitzler, G.R., Kingston, R.E., and Schreiber, S.L. (1998). Chromatin deacetylation by an ATP-dependent nucleosome remodelling complex. Nature *395*, 917–921.

Wade, P.A., Jones, P.L., Vermaak, D., and Wolffe, A.P. (1998). A multiple subunit Mi-2 histone deacetylase from *Xenopus laevis* cofractionates with an associated Snf2 superfamily ATPase. Curr. Biol. *8*, 843–846.

Wiechens, N., Singh, V., Gkikopoulos, T., Schofield, P., Rocha, S., and Owen-Hughes, T. (2016). The chromatin remodelling enzymes SNF2H and SNF2L position nucleosomes adjacent to CTCF and other transcription factors. PLoS Genet. *12*, e1005940.

Wu, T.D., and Nacu, S. (2010). Fast and SNP-tolerant detection of complex variants and splicing in short reads. Bioinformatics *26*, 873–881.

Xue, Y., Wong, J., Moreno, G.T., Young, M.K., Côté, J., and Wang, W. (1998). NURD, a novel complex with both ATP-dependent chromatin-remodeling and histone deacetylase activities. Mol. Cell *2*, 851–861.

Zhang, Y., LeRoy, G., Seelig, H.-P., Lane, W.S., and Reinberg, D. (1998). The dermatomyositis-specific autoantigen Mi2 is a component of a complex containing histone deacetylase and nucleosome remodeling activities. Cell *95*, 279–289.

Zhang, H., Gao, L., Anandhakumar, J., and Gross, D.S. (2014). Uncoupling transcription from covalent histone modification. PLoS Genet. *10*, e1004202.

Zhang, W., Aubert, A., Gomez de Segura, J.M., Karuppasamy, M., Basu, S., Murthy, A.S., Diamante, A., Drury, T.A., Balmer, J., Cramard, J., et al. (2016). The nucleosome remodeling and deacetylase complex NuRD is built from preformed catalytically active sub-modules. J. Mol. Biol. *428*, 2931–2942.

STAR★METHODS

KEY RESOURCES TABLE

REAGENT or RESOURCE	SOURCE	IDENTIFIER
**Antibodies**		
Chd4, mouse monoclonal	Abcam	RRID:AB_2229454
ER, rabbit polyclonal	Santa Cruz Biotechnology	RRID:AB_631471
Esrrb, mouse monoclonal	R&D SYSTEMS	RRID:AB_2100412
GATAD2A, rabbit monoclonal	Abcam	RRID:AB_1952305
GATAD2B, rabbit polyclonal	Bethyl Labs	RRID:AB_937934
H3, rabbit polyclonal	Abcam	RRID:AB_302613
H3 K27ac, rabbit polyclonal	Abcam	RRID:AB_2118291
H3 K4me3, rabbit polyclonal	Millipore	RRID:AB_1163444
Hdac1, rabbit polyclonal	Abcam	RRID:AB_305705
Hdac2, rabbit polyclonal	Santa Cruz	RRID:AB_2118563
Klf4, goat polyclonal	R&D Systems	RRID:AB_2130245
LaminB1, rabbit polyclonal	Abcam	RRID:AB_2616597
Mbd3, rabbit monoclonal	Abcam	ab157464
Mbd3, rabbit polyclonal	Bethyl Labs	RRID:AB_1998980 BATCH NUMBER 1
Med1, rabbit polyclonal	Bethyl Labs	RRID:AB_577241
Med12, rabbit polyclonal	Bethyl Labs	RRID:AB_669756
Mta1, rabbit monoclonal	Cell Signaling	RRID:AB_10705601
Mta1/2, goat polyclonal	Santa Cruz	RRID:AB_649541
Mta2, mouse monoclonal	Abcam	RRID:AB_2146939
Mta3, rabbit polyclonal	Proteintech	RRID:AB_2298003
Nanog, rabbit polyclonal	Bethyl Labs	RRID:AB_386108
P300, rabbit	Santa Cruz	RRID:AB_2616339
PCNA, mouse monoclonal	Santa Cruz	RRID:AB_628110
Pou5f1, goat polyclonal	Santa Cruz	RRID:AB_653551
Rbbp4, rabbit monoclonal	Abcam	RRID:AB_1603754
RNA Polymerase II CTD4H8 (SER 5P), mouse monoclonal	Millipore	RRID:AB_309852
RNA Polymerase II N-20 (Polr2a), rabbit	Santa Cruz	sc-899 X
RNA Polymerase II NTD D8L4Y (Polr2a), rabbit monoclonal	Cell Signaling	RRID:AB_2687876
Sin3a, rabbit polyclonal	Santa Cruz	RRID:AB_2187760
**Chemicals, Peptides, and Recombinant Proteins**		
Trizol	Life Technologies	15596018
Micrococcal nuclease	New England Biolabs	M0247S
4-thiouridine	Sigma	T4509
4-hydroxytamoxifen	Sigma	H7904
Mouse ES cell line: Mbd3-3xFLAG knock-in/homozygous floxed	BH Lab	BHA
Mouse ES cell line: Mbd3$^{\Delta/\Delta}$	BH Lab	BHAKO
**Deposited Data**		
Mbd3 Inducible ES cells, time 0, 0.5, 4, 24h: 48h Input	This paper	E-MTAB-6804
Mbd3 Inducible ES cells, time 0, 0.5, 4, 24h: Nanog ChIP	This paper	E-MTAB-6804
Mbd3 Inducible ES cells, time 0, 0.5, 4, 24h: Klf4 ChIP	This paper	E-MTAB-6804
Mbd3 Inducible ES cells, time 0, 0.5, 4, 24h: Med12 ChIP	This paper	E-MTAB-6804
Mbd3 Inducible ES cells, time 0, 0.5, 4, 24h: RNA Polymerase II S5P ChIP	This paper	E-MTAB-6804
Mbd3 Inducible ES cells, time 0, 24h, 48h: Chd4 ChIP	This paper	E-MTAB-6804
Mbd3 Inducible ES cells, time 0, 24h, 48h: FLAG ChIP	This paper	E-MTAB-6804

(*Continued on next page*)

Continued

REAGENT or RESOURCE	SOURCE	IDENTIFIER
Mbd3 Inducible ES cells, time 0, 1h, 4h, 8h, 24h, 48h: H3K27Ac ChIP	This paper	E-MTAB-6804
Mbd3 Inducible ES cells, time 0, 1h, 4h, 8h, 24h: H3K4Me3 ChIP	This paper	E-MTAB-6804
Mbd3 Inducible ES cells, time 0, 1h, 4h, 8h, 24h, 48h: H3 ChIP	This paper	E-MTAB-6804
$Mbd3^{FLAG/-}$ Input	This paper	E-MTAB-6804
$Mbd3^{FLAG/-}$ Chd4 ChIP	This paper	E-MTAB-6804
$Mbd3^{FLAG/-}$ FLAG ChIP	This paper	E-MTAB-6804
$Mbd3^{FLAG/-}$ Klf4 ChIP	This paper	E-MTAB-6804
$Mbd3^{FLAG/-}$ Nanog ChIP	This paper	E-MTAB-6804
$Mbd3^{-/-}$ Input	This paper	E-MTAB-6804
$Mbd3^{-/-}$ Chd4 ChIP	This paper	E-MTAB-6804
$Mbd3^{-/-}$ Klf4 ChIP	This paper	E-MTAB-6804
$Mbd3^{-/-}$ Nanog ChIP	This paper	E-MTAB-6804
Mbd3 Inducible ES cells, time 0, 0.5, 1, 4, 24, 48h nascent RNA seq	This paper	E-MTAB-6805
Mbd3 Inducible ES cells, time 0, 0.5, 1, 4, 24, 48h mRNA seq	This paper	E-MTAB-6806
Mbd3 Inducible ES cells, time 0, 0.5, 24h MNase-seq	This paper	E-MTAB-6807
Other		
WT ES RNA-seq dataset	PMID: 27471257	E-MTAB-4566
$Mbd3^{-/-}$ ES RNA-seq dataset	PMID: 27471257	E-MTAB-4566
H3K27Me3 ChIP-seq dataset	PMID: 22541430	GSE23943
H3K36Me3 ChIP-seq dataset	PMID: 22541430	GSE23943
H3K9Me3 ChIP-seq dataset	PMID: 22541430	GSE23943
H3K27Ac ChIP-seq dataset	PMID: 24905168	GSE56138
H3K4Me1 ChIP-seq dataset	PMID: 24905168	GSE56138
H3K4Me3 ChIP-seq dataset	PMID: 24905168	GSE56138
Ezh2 ChIP-seq dataset	PMID: 22541430	GSE23943
Esrrb ChIP-seq dataset	PMID: 27471257	E-MTAB-4565
Nanog ChIP-seq dataset	PMID: 27471257	E-MTAB-4565
Oct4 ChIP-seq dataset	PMID: 27471257	E-MTAB-4565
Klf4 ChIP-seq dataset	PMID: 27471257	E-MTAB-4565
EP300 ChIP-seq dataset	PMID: 24905168	GSE56138

CONTACT FOR REAGENT AND RESOURCE SHARING

Further information and requests for resources and reagents should be directed to and will be fulfilled by the lead contact, Brian Hendrich (Brian.Hendrich@cscr.cam.ac.uk, @BDH_Lab).

EXPERIMENTAL MODEL AND SUBJECT DETAILS

Tissue culture

Mouse embryonic stem (ES) cells were cultured in 2i/LIF (2iL) media on gelatin-coated plates unless otherwise specified. *Mbd3* conditional and null ES cell lines have been described (Kaji et al., 2006; Stevens et al., 2017) and were created in an E14Tg2a (XY) background. Translocation of Mbd3b protein to the nucleus was induced with 4-hydroxytamoxifen added directly to the culture media to a final concentration of 0.4 nM for varying times as indicated. Alkaline phosphatase assays were performed by plating 1000 cells into a 6-well plate and expanding for 4 days prior to staining according to the manufacturer's protocol (Sigma). N = 3 to 12 wells per condition. Colonies were scored blind to genotype.

METHOD DETAILS

Immunoprecipitation and western blotting

Immunoprecipitation and western blotting were carried out using standard methods. Antibodies and concentrations used are listed in Table S1. All original, uncropped blots are available at Mendeley Data: https://doi.org/10.17632/2whjxyxyc9.1

ChIP, ChIP-seq and ChIP-seq data analyses

Chromatin immunoprecipitation (ChIP) was performed using standard protocols. Fixation was carried out using 1% formaldehyde 10 minutes at room temperature and quenched with 150 mM glycine. DNA was fragmented in the presence of 0.4% SDS using a Bioruptor sonication instrument (Diagenode) producing a size range of 200 to 300 bp. Antibodies used are listed in Table S1. Locus-specific primers used for quantitative PCR are listed in Table S2. ChIP-seq libraries were prepared using the NEXTflex Rapid DNA-seq kit (Illumina) and sequenced at the CRUK Cambridge Institute Genomics Core facility (Cambridge, UK) on the Illumina platform.

ChIP-seq libraries were filtered for adaptor sequences and aligned to the mouse reference genome (mm10/GRCm38) using Bowtie (v1.0.1/1.1.1) (Langmead et al., 2009) with parameters -y –best–strata–nomaqround and filtered for uniquely mapped reads (-m 1). Duplicate reads were removed with Picard tools (v1.114/1.76). Enriched regions (or peaks) for Mbd3, Chd4 and transcription factors were called with MACS2 (v2.1.0.20140616/20150420) (Liu, 2014) with default parameters using DNA input as control and retaining all statistically enriched regions (FDR < 1%). ChIP-seq peaks were annotated relative to Ensembl genomic features with PeakAnalyzer (Salmon-Divon et al., 2010). NuRD peaks were defined as the union of Mbd3 and Chd4 peaks which overlap by at least 1bp.

Klf4 and Nanog regions that were differentially bound in wild-type and *Mbd3*$^{-/-}$ cells were identified using diffBind (Stark and Brown, 2017). First, a set of consensus peaks found in at least two replicates was defined. Read counts were then calculated across those peaks and input counts subtracted, followed by differential analysis reporting the significantly bound regions (FDR < 0.05).

Regions enriched for histone modifications were detected relative to corresponding sequenced input-DNA or H3 controls using MACS2 with broad peak mode at FDR threshold of 5%. Where biological replicates were available, consensus peaks were identified in the pooled dataset (obtained by pooling reads from all replicates). Peaks were filtered for regions blacklisted by ENCODE.

Values for read densities were adjusted by subtracting those found in the corresponding input experiment normalized for sequencing depth with deeptools (v1.6.0). We calculate ChIP-seq enrichment in 10bp bins as Reads per Genomic Content (RPGC).

Regions enriched for H3K4me1 were categorized into inactive enhancers (without H3K4me3 enrichment) or active enhancers (with overlapping H3K27ac and p300 enrichment). If more than one published dataset was available for a given histone modification or p300, only peaks called in all datasets were considered in enhancer identification.

Traveling ratio was calculated for all genes in Ensembl 75, defined as the ratio of RNA Polymerase II Serine 5 phosphorylation density (RPKM) in promoters to that in the gene body and promoter (Figure 6C). The promoter-proximal region is defined using a fixed window of −2Kb to 500bp around the gene start. The gene body is from +501bp to the gene end. Only genes with at least 1 RPKM reads in the gene promoter and body were included in these analyses.

RNA-seq and expression analysis

Metabolic labeling of cells for purification of nascent transcript was carried out as described (Rädle et al., 2013). Cells were treated with tamoxifen for the times indicated and pulse labeled with 500 μM 4-thiouridine for 15 minutes prior to harvesting in Trizol (Life Technologies) for RNA purification. For isolation of total RNA for sequencing, cells were harvested in Trizol and processed according to manufacturer's instructions.

Libraries for sequencing were prepared using the NEXTflex Rapid Directional RNA-seq kit (Illumina) or SMARTer Stranded Total RNA-Seq Kit v2 - Pico Input Mammalian (Takara Bio) and sequenced as above. Individual expression assays were carried out using gene-specific TaqMan probes (Life Technologies).

RNA-seq libraries were mapped against the mouse reference genome mm10 using GSNAP (gmap-2014- 12-17) (Wu and Nacu, 2010) with parameters "–m 7 –i 1 –N 1 –w 100000 –E 100 –n 10." Gene read counts were calculated with HTSeq (v0.6.1) based on gene annotation from Ensembl release 75, and normalization and differential expression analysis were performed using the R package DESeq2 (Love et al., 2014) with the default model. We identified differentially expressed genes at FDR-adjusted *p*-values less than 0.05. For time series analyses differential expression was assessed for all pairwise comparisons against the 0 h time point.

MNase-seq and MNase qPCR

For nucleosome positioning analyses, cells were grown in 2iL conditions (supplemented with tamoxifen as noted). Cells were harvested, washed in ice cold PBS and resuspended in ice cold lysis buffer (10mM Tris-HCl pH7.4, 10mM NaCl, 3mM $MgCl_2$, 0.5% IGEPAL, 150mM spermine and 500mM spermidine) at a concentration of 10^6 cells/ml. 1ml aliquots were centrifuged at 300 × *g* for 10 min at 4°C and pellets washed in 1ml digestion buffer (10mM Tris-HCl pH7.4, 15mM NaCl, 60mM KCl, 150mM spermine and 500mM spermidine). MNase digestion was carried out in 100ul digestion buffer containing 1mM CaCl and 2000U/ml micrococcal nuclease (New England Biolabs) at 24°C for 15min with shaking. The reaction was terminated using an equal volume of stop buffer (digestion buffer containing 20mM EDTA, 2mM EGTA) before RNase and Proteinase K treatment. Monosomal DNA was isolated by phenol chloroform extraction followed by gel purification and either used for library preparation in the case of MNase-seq or for quantitative PCR. Locus-specific primers and their positions relative to annotated transcription start sites are listed in Table S2.

MNase-seq data were filtered for adaptor sequences and low-quality bases using Trim Galore with default parameters. Reads were aligned to the mouse reference genome GRCm38/mm10 using Bowtie (Langmead et al., 2009) with parameters "-y -m 1 –maxins 300 –allow-contain –nomaqround." The aligned reads were sorted and duplicates were removed using Picard tools (v1.114).

ChIP-seq binding sites for CTCF were obtained from the CODEX project (Sánchez-Díaz et al., 2001). Additional published ChIP-seq data referenced in this study are listed in Table S3.

Aggregate profiles of MNase-seq, Nascent RNA-seq and ChIP-seq

Aligned reads were converted to bigwig format at single-nucleotide resolution and normalized to uniform mean coverage using deeptools (Ramírez et al., 2016) bamCoverage with parameters "–binSize 1–normalizeTo1x 2150570000" and using the "–blackListFileName" command on the mouseENCODE blacklisted regions (ENCODE Project Consortium, 2012) for reference assembly GRCm38/mm10. Additionally, the option "–centerReads" was used on single end data and "–extendReads" was applied to all samples, with fragment length specified as 200 for transcription factor ChIP-seq and 250 for PolII ChIP-seq.

Aggregate profile data across different features were extracted from bigwig files using deepTools computeMatrix with parameters "–binSize 5 -b 1500 -a 1500 –missingDataAsZero" and using a bed file with the coordinates of the selected feature. The option "–referencePoint tss" was used for the tss coordinates and "–referencePoint center" for all other features. The resulting MNase-seq and ChIP-seq data were first normalized by the mean signal at each individual location. The aggregate signal for across all locations per replicate was further normalized by dividing it by the mean signal at flanking positions −1500 to −1450 and +1450 to +1500, to yield enrichment over baseline using a consistent baseline across all replicates. Nascent RNA-seq data were not treated with these normalization steps. The final aggregate profiles display the mean of the three biological replicates. A 95% confidence interval of the MNase-seq and ChIP-seq mean was calculated by bootstrapping, with 5000 iterations for each biological sample replicate and with the normalization procedure repeated independently. A 95% confidence interval of the nascent RNA-seq mean was calculated as ± 1.96 s.e.m. Empirical p values were computed based on the bootstrap replicates.

Transcription start site coordinates were extracted from Gencode vM9.

QUANTIFICATION AND STATISTICAL ANALYSIS

Data analysis and statistical tests for qPCR data were performed using GraphPad Prism, and p values calculated using a two-tailed t test. P values for high throughput sequencing data were calculated using bootstrapping.

DATA AND SOFTWARE AVAILABILITY

Sequencing data are available in the ArrayExpress repository under accessions E-MTAB-6804 (ChIP-seq), E-MTAB-6805 (nascent RNA-seq), E-MTAB-6806 (total RNA-seq) and E-MTAB-6807 (MNase-seq).

Molecular Cell, Volume *71*

Supplemental Information

The Nucleosome Remodeling and Deacetylation Complex Modulates Chromatin Structure at Sites of Active Transcription to Fine-Tune Gene Expression

Susanne Bornelöv, Nicola Reynolds, Maria Xenophontos, Sarah Gharbi, Ewan Johnstone, Robin Floyd, Meryem Ralser, Jason Signolet, Remco Loos, Sabine Dietmann, Paul Bertone, and Brian Hendrich

Bornelöv et al. Figure S1

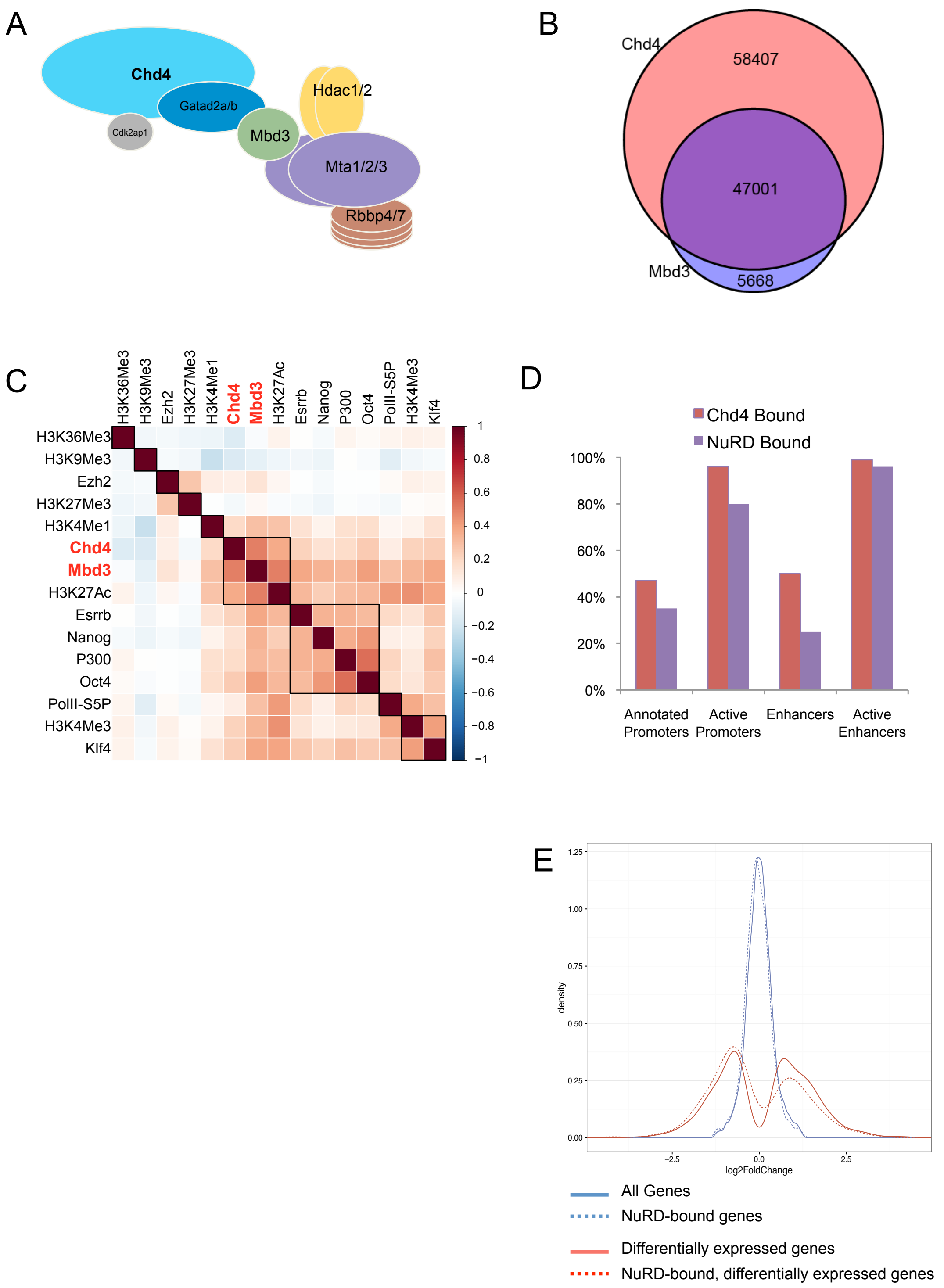

Figure S1. NuRD modulates active transcription. (Related to Figure 1)

A. Schematic of the NuRD complex. Proteins are indicated at approximate stoichiometry. Mbd3 acts to hold together the remodelling subcomplex, containing

Chd4, and the histone deacetylase-containing subcomplex.

B. Overlap of peaks called from Mbd3 or Chd4 ChIP-seq data (blue and pink circles, respectively) in mouse ES cells. Numbers of peaks corresponding to Chd4-only, Mbd3 and Chd4, or Mbd3-only are indicated.

C. Correlation between ChIP peaks for the indicated histone modifications and transcription factors in 2i/LIF. Datasets are ordered by unsupervised clustering. Boxes indicate the highest correlations. Datasets used are listed in the STAR Methods.

D. The proportion of promoters and enhancers containing a Chd4- or NuRD-bound (defined as Mbd3- and Chd4-bound) site are plotted.

E. Changes in gene expression in *Mbd3*-null vs wild-type ES cells. Red lines indicate genes that exhibit a significant change in mutant cells, blue lines indicate no significant change. Genes showing a ChIP-seq peak for Mbd3 and Chd4 between -2 Kb and + 0.5 Kb of the annotated TSS are indicated as dotted lines. y-axis: kernel density estimation; x-axis: $\log_2$ fold change (KO/WT).

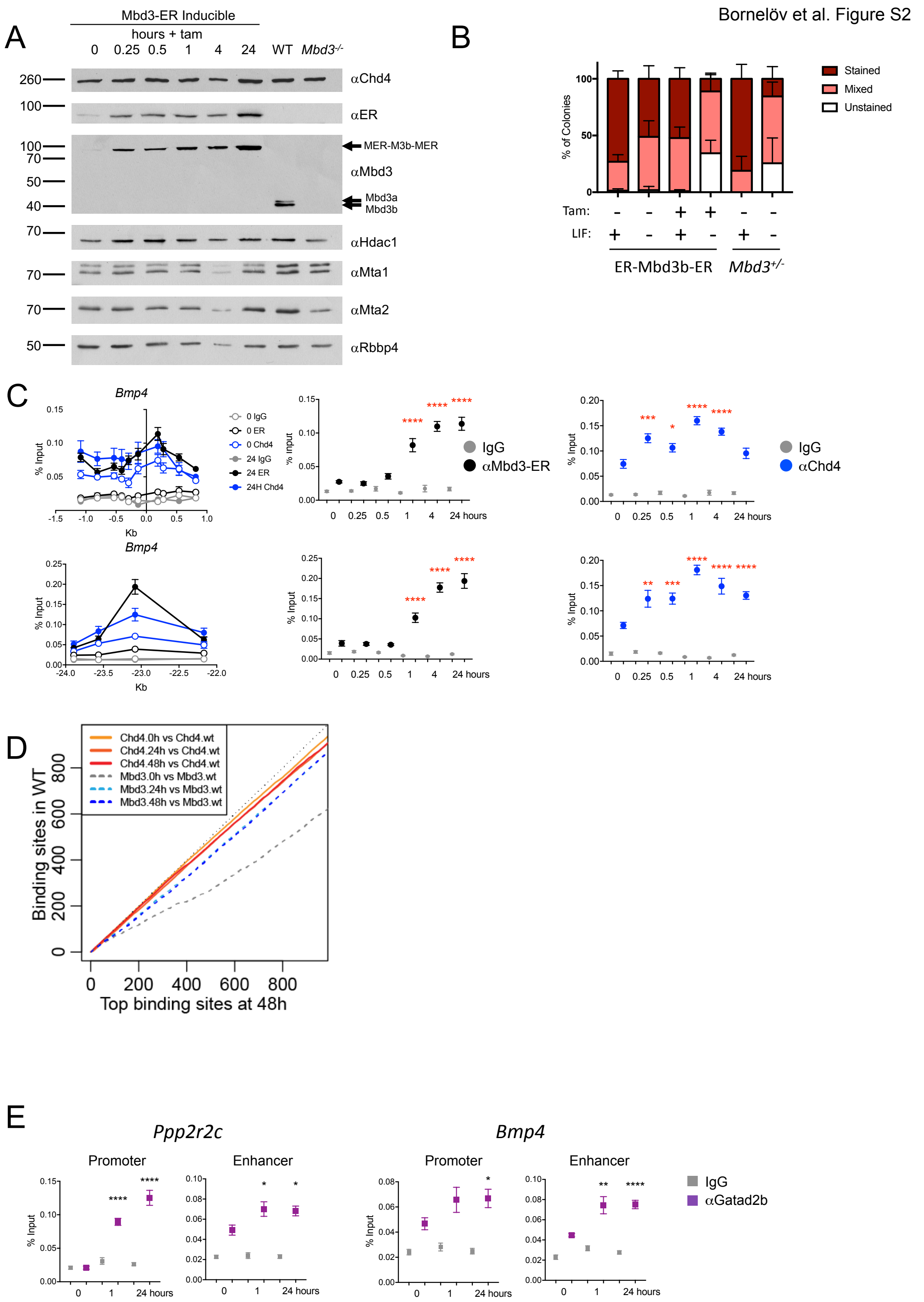

Figure S2. Further characterisation of the Mbd3 inducible system (Related to Figure 1).

A. Nuclear extracts obtained from Mbd3 inducible ES cells at different times after tamoxifen addition, from wild type ES cells (WT), or from *Mbd3*-null ES cells (*Mbd3*$^{-/-}$)

were probed with antibodies indicated at right. In the α-Mbd3 panel the location of the Mer-Mbd3b-Mer transgene is indicated with an arrow, as are the locations of endogenous Mbd3 isoforms in wild type cells. Protein sizes are shown at left in kilodaltons.

B. Mbd3b inducible cells with or without tamoxifen (+ or - TAM) and Mbd3(+/-) ES cells were plated in self-renewing (Serum+LIF) or differentiation conditions (Serum without LIF) at clonal density and allowed to grow for five days prior to staining for alkaline phosphatase activity and scored blind. The percentage of unstained, mixed, or fully stained colonies is shown. Mean ± SD is plotted where N = 3-12 per condition.

C. Left: ChIP-qPCR for Mbd3 (black lines), Chd4 (blue lines) and IgG control (grey lines) across the promoter (top) and an enhancer (bottom) for *Bmp4*. N ≥ 3 biological replicates. X-axes show locations relative to the annotated transcription start site of *Bmp4*. ChIP-qPCR at the peak of the Mbd3 ChIP signal was plotted across a time course of tamoxifen addition (middle and right panels). **** P<0.0001, *** P<0.001, ** P<0.01, * P<0.05) using a two-tailed t-test. N ≥ 3 biological replicates.

D. Validation of Mbd3 and Chd4 binding site recovery after Mbd3 induction in the inducible cell line. The cumulative overlap between binding sites 0h, 24h, or 48h after Mbd3 induction and in WT is shown. All binding sites were ranked by their p-value and the 20,000 most significant sites in WT were considered for the overlap calculations.

E. ChIP-qPCR for Gatad2b across the tamoxifen induction time course at single points at the indicated features. **** P<0.0001, *** P<0.001, ** P<0.01, * P<0.05) using a two-tailed t-test. N ≥ 3 biological replicates.

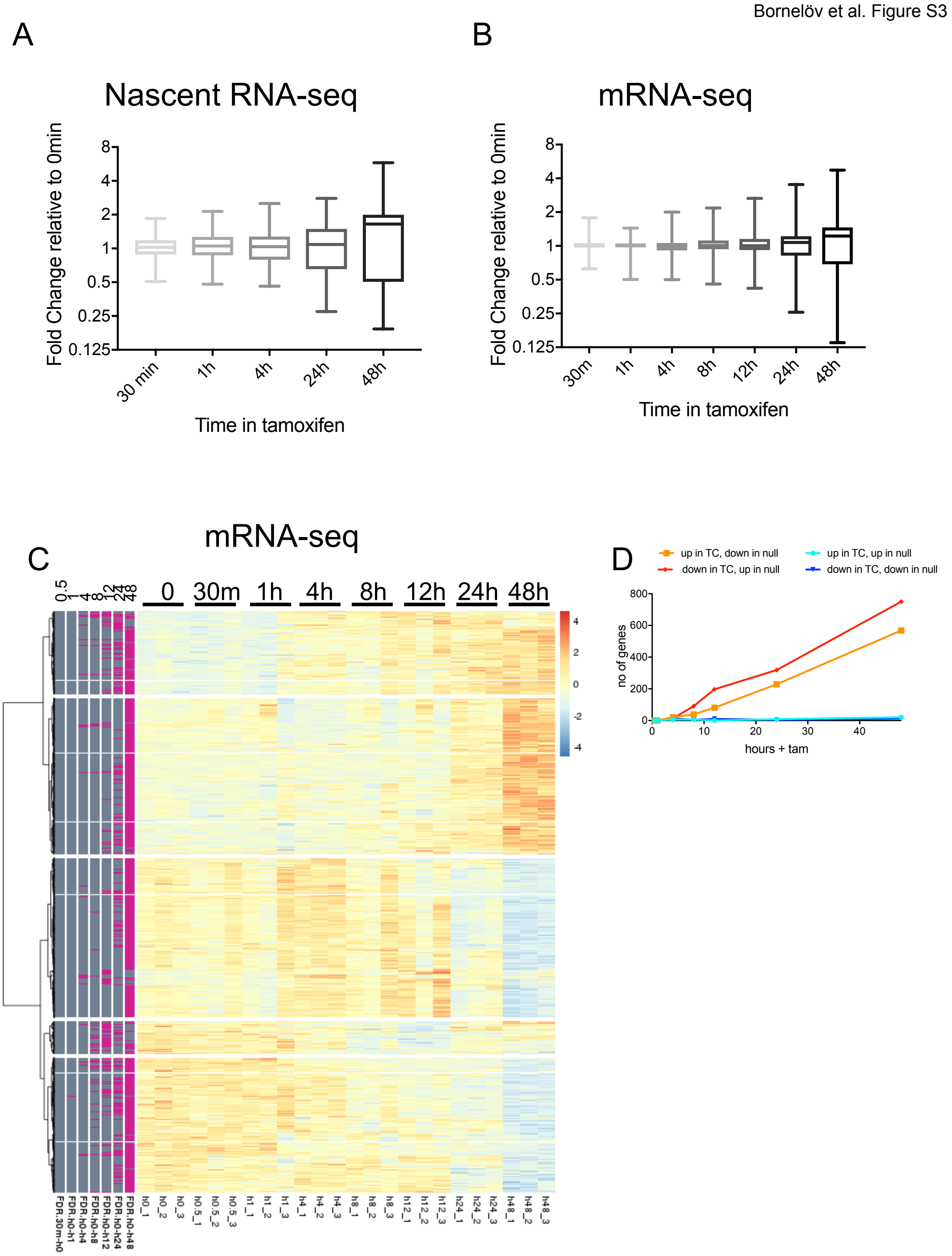

Figure S3. Gene expression changes in the Mbd3 inducible system (Related to Figure 1).

A and B: Fold change in gene expression plotted for all genes showing significant change (P<0.01, |FC|>2) at 48 hours of tamoxifen treatment relative to untreated cells for nascent RNA-seq (A; N = 816) and for mRNA-seq (B; N = 2210).

C. Unsupervised clustering of gene expression changes over indicated times of the time course of tamoxifen exposure as measured by mRNA-seq. Data for each of three biological replicates are shown. Genes exhibiting a significant change relative to time 0 are shaded pink in the columns at left.

D Comparison of changes in gene expression in the Mbd3 induction time course with changes seen in steady-state Mbd3-null ES cells.

Bornelöv et al. Figure S4

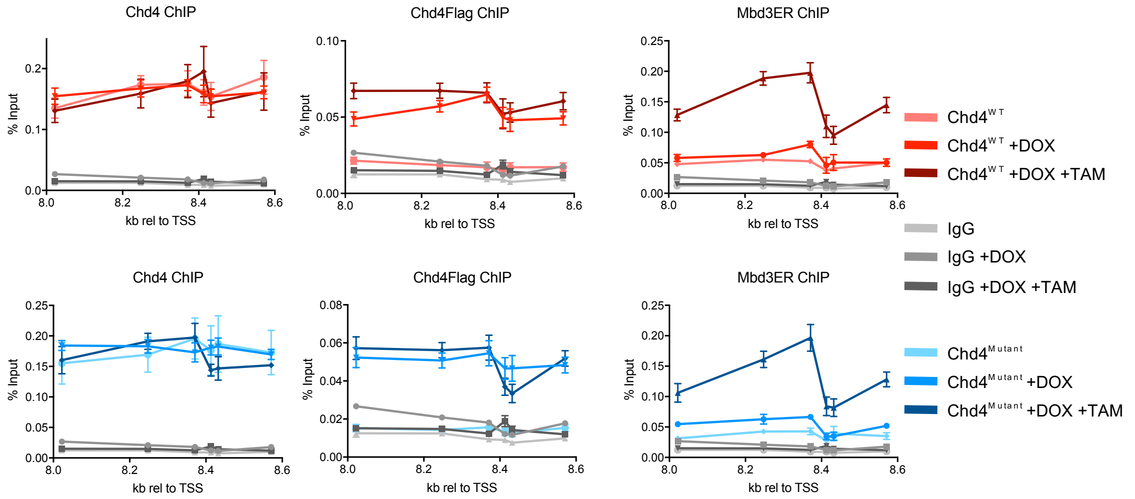

Figure S4. Induced Chd4 protein associates with chromatin (Related to Figure 4).

ChIP-qPCR across the *Ppp2r2c* enhancer for Chd4 (left), anti-FLAG, which recognises the Chd4 transgenic protein (middle) and for the induced Mbd3 protein (right) in ES cells harbouring the wild type Chd4 transgene (Chd4^{WT}) or the ATPase mutant Chd4 transgene (Chd4^{Mutant}). ChIP was performed in uninduced cells and in cells induced with DOX only or DOX and TAM. N = 3-6 biological replicates.

Bornelöv et al. Figure S5

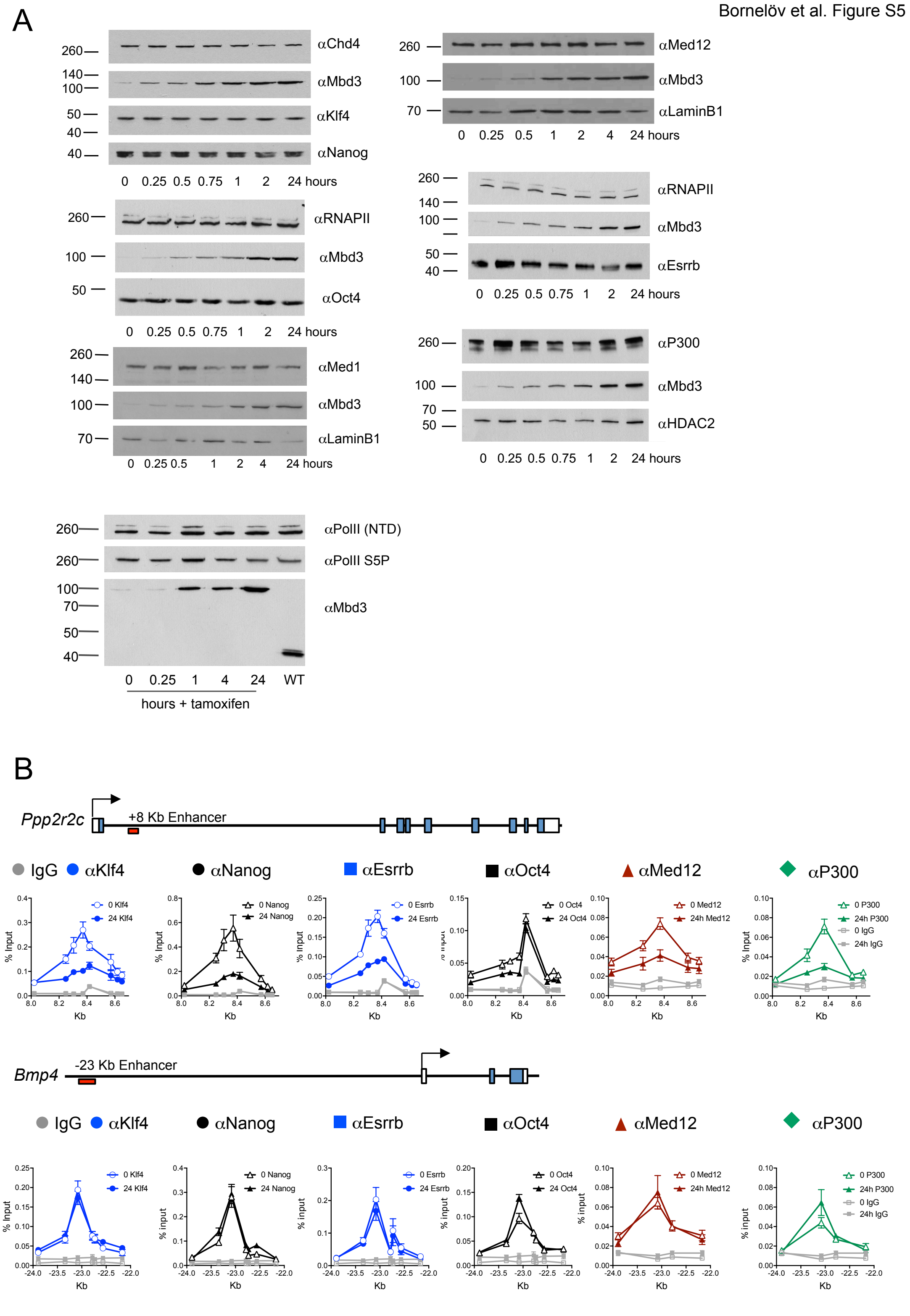

Figure S5. Transcription factors behaviour across the Mbd3 induction time course (Related to Figure 5).

A. Nuclear extracts obtained from Mbd3 inducible ES cells at different times across a tamoxifen addition time course were probed with indicated antibodies. Each set of panels was taken from the same gel. Protein sizes are shown at left in kilodaltons. Full western blot images are available in Supplemental Data.

B. ChIP-qPCR for indicated proteins across the *Ppp2r2c* enhancer or the *Bmp4* enhancer at 0 or 24 hours of tamoxifen exposure. A schematic of each gene is shown above the ChIP-qPCR panels. Mean ± SEM is plotted for all points. N ≥ 3 biological replicates.

Bornelöv et al. Figure S6

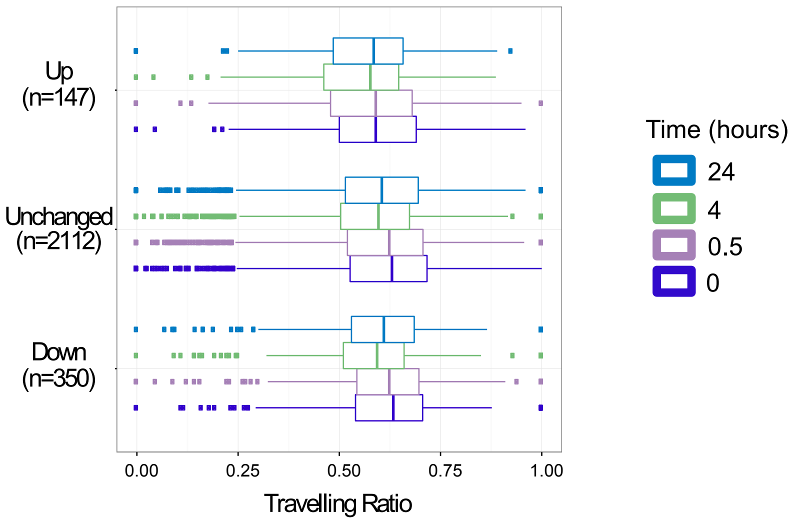

Figure S6. Travelling ratios as a function of changes in gene expression (Related to Figure 6).

Travelling ratios for genes showing increased (Up), decreased (Down) or no change (Unchanged) in the Mbd3 induction time course are plotted at indicated times after tamoxifen addition.
